# Alternative Splicing: Molecular Mechanisms, Biological Functions, Diseases, and Potential Therapeutic Targets

**DOI:** 10.1002/mco2.70545

**Published:** 2025-12-14

**Authors:** Zhi‐Min Zhu, Xiao‐Mei Wu, Yan Hu, Xiao‐Lan Bian, Ya‐Qin Wang, Qiong‐Ni Zhu

**Affiliations:** ^1^ Department of Pharmaceutics Shanghai Eighth People's Hospital Shanghai China; ^2^ Department of Pharmacy Ruijin Hospital Shanghai Jiao Tong University School of Medicine Shanghai China; ^3^ Clinical Laboratory Center The First Hospital of Hunan University of Chinese Medicine Changsha Hunan China; ^4^ Department of Pharmacy RuiJin Hospital Lu Wan Branch Shanghai Jiao Tong University School of Medicine Shanghai China; ^5^ Department of Pharmacy Henan Provincial People's Hospital People's Hospital of Zhengzhou University School of Clinical Medicine Henan University Zhengzhou Henan China

**Keywords:** alternative splicing, biological functions, diseases, molecular mechanisms, therapeutic targets

## Abstract

Alternative splicing (AS) is an important posttranscriptional process that increases proteomic complexity of eukaryotes. Through the selective inclusion or exclusion of exons, AS fine‐tunes gene expression and underpins diverse biological processes. Recent research revealed that AS is controlled not only by spliceosomal components but also by dynamic RNA structures and the spatial compartmentalization of splicing factors within biomolecular condensates formed via liquid–liquid phase separation (LLPS). Nevertheless, a unified framework connecting these mechanistic insights with emerging therapeutic strategies remains lacking. This review systematically integrates current knowledge of AS regulation, encompassing the architecture and dynamics of the core spliceosome, structural RNA elements such as G‐quadruplexes, and LLPS‐driven condensates exemplified by oncogenic SRSF9 droplets. It further delineates how AS influences cell development, immune modulation, and stress adaptation, while its dysregulation contributes to human pathologies, including SF3B1 mutant cancers, TDP‐43‐associated neurodegeneration, and cardiovascular disease. We critically appraise therapeutic innovations targeting aberrant splicing, including small molecule spliceosome modulators, antisense oligonucleotides like nusinersen, and CRISPR/dCas13‐based RNA editing. By integrating molecular mechanisms with translational advances, this review provides a conceptual framework to accelerate RNA‐targeted precision medicine in the era of spatial multiomics and artificial intelligence.

## Graphical abstract

Alternative splicing (AS) expands proteomic diversity and functional complexity in eukaryotes, regulated by spliceosomal components, RNA elements, and epigenetic modifications. Dysregulated AS contributes to diseases, including cancer, neurodegenerative disorders, and cardiovascular conditions, among others. Therapeutic interventions, such as antisense oligonucleotides and small molecule modulators, aim to correct splicing defects and restore isoform balance for precision medicine.

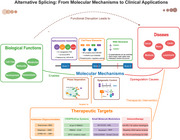

## 1 Introduction

The central dogma of molecular biology delineates the traditional pathway of genetic information transfer from DNA to RNA and subsequently to protein, forming the foundational basis of our comprehension of biological systems [1]. Nevertheless, the simplistic concept that one gene encodes one protein has been fundamentally challenged. This is primarily attributed to the remarkable complexity of the eukaryotic transcriptome, with alternative splicing (AS) playing a pivotal role [2]. As an essential posttranscriptional regulatory mechanism, AS permits a single gene to generate multiple mRNA splice variants and protein isoforms, thereby significantly enhancing the functional diversity and regulatory precision of the proteome, despite a finite number of genes [3]. AS functions as a central molecular mechanism driving embryonic development, tissue‐specific functions, and cellular differentiation [4].

Fundamentally, AS involves the selective inclusion or exclusion of exons, and occasionally the retention of introns, during pre‐mRNA processing, resulting in distinct mature transcripts [5]. This intricately regulated process is controlled by dynamic interactions between cis‐regulatory elements, such as exonic/intronic splicing enhancers or silencers, and trans‐acting factors, including SR and hnRNPs proteins [6]. The resultant protein isoforms often differ significantly in terms of stability, subcellular localization, and function [7]. Therefore, AS is not just a processing step, it is a basic and ubiquitous biological mechanism that regulates gene expression, dictates cell fate, and influences almost every essential process, ranging from immune responses and neural transmission to cell cycle control.

Dysregulation of splicing is intimately linked to the pathophysiology of numerous human diseases because of its essential function in maintaining cellular homeostasis [8, 9, 10]. Defective or toxic protein isoforms can result from splice sites or regulatory elements, abnormal expression of core spliceosomal components or splicing factors, and mistakes in splice site selection [11]. Pathogenic splicing events constitute a prevalent etiological factor across various conditions, including cancer [8, 9], neurodegenerative disorders like Alzheimer's disease (AD) [12, 13] and amyotrophic lateral sclerosis (ALS) [14], as well as rare genetic diseases [15]. This increasing knowledge is rapidly changing the spliceosome and its regulatory network from basic biological concepts into potential therapeutic targets. New technologies like antisense oligonucleotides (ASO) and small molecule splicing modulators (SMSMs) make it possible to targeted correction of pathological splicing or selective inhibition of cancer‐associated variants. This marks the start of a new era of RNA‐centered precision medicine.

This review aims to provide a comprehensive overview of AS. We commence with an examination of the molecular mechanisms that govern splicing accuracy and plasticity, subsequently investigating its vital biological functions within physiological frameworks. We then summarize human diseases caused by splicing defects, illustrating with representative examples. The review highlights recent advances in splicing‐targeted therapeutic strategies, discussing both their immense potential and persistent challenges. By integrating these perspectives, this review offers a complete picture of AS, showing it as both a basic biological process and one of the most exciting and cutting‐edge fields in biomedical research and drug development.

## 2 Molecular Mechanisms

AS significantly expands the proteomic diversity of eukaryotes by generating multiple mRNA isoforms from a single gene, thereby improving cellular adaptability [5]. This highly coordinated process involves the core spliceosome, dynamic signaling networks, and adaptations that are specific to the situation, like oncogenic isoform switching [6]. Understanding the molecular basis is crucial for elucidating the functional roles of AS in both health and disease.

The multilayered molecular machinery that controls AS is explored in this section. The precise recognition by cis‐elements and trans‐factors, core spliceosome components and their functions, the regulatory role of RNA structures like G‐quadruplexes, and the arrangement of splicing factors within biomolecular condensates via liquid–liquid phase separation (LLPS) are all described. It also explores the integration of epigenetic, cotranscriptional, and environmental signals, concluding with advances in high‐throughput technologies for splicing analysis.

### 2.1 Core Regulatory Components of AS

The precision of AS relies on the coordinated action of the spliceosome and its associated regulatory factors [16]. The five main small nuclear ribonucleoproteins (U1, U2, U4, U5, and U6 snRNPs) and more than 200 auxiliary proteins make up the spliceosome, a large, dynamic ribonucleoprotein complex [17, 18]. These core elements are crucial for precise splicing site selection in addition to catalyzing the splicing reaction [17, 19].

**FIGURE 1:**
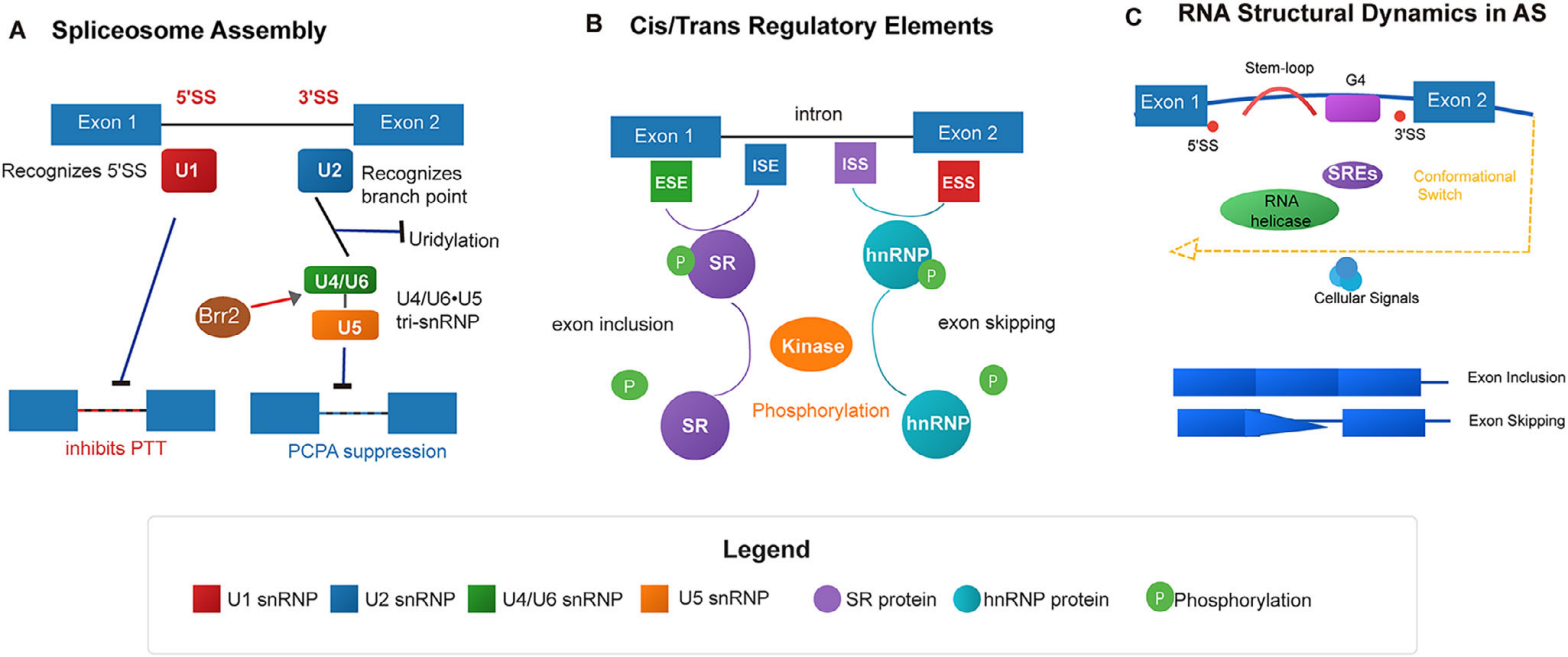
Dynamic assembly of the spliceosome and its core regulatory mechanisms. (A) Stepwise assembly of major snRNPs (U1, U2, U4/U6•U5) onto pre‐mRNA, illustrating their roles in recognizing splice sites and suppressing premature transcription termination (PTT) and cleavage/polyadenylation (PCPA). U1 snRNP base‐pairs with the 5′ splice site (5′SS) and inhibits intronic PCPA. The U4/U6•U5 tri‐snRNP is activated by the Brr2 helicase, and U6 prevents transcript uridylation by TUT1. (B) The combinatorial interplay between cis‐regulatory elements (exonic splicing enhancers, ESEs; exonic splicing silencers, ESSs; intronic splicing enhancers, ISEs; intronic splicing silencers, ISSs) and trans‐acting factors (e.g., SR proteins and hnRNPs) determines exon inclusion or skipping. Cell‐specific signals, such as kinase‐mediated phosphorylation, fine‐tune splicing decisions by modulating the activity of these factors. (C) RNA secondary structures (e.g., stem‐loops, G‐quadruplexes, G4s) act as conformational switches that modulate splicing efficiency by occluding or exposing splice sites (SS) or splicing regulatory elements (SREs). RNA helicases (e.g., DHX36) and ATP‐dependent remodelers (e.g., Brr2) resolve or stabilize these structures in response to cellular cues.

As summarized in Table 1 and Figure 1A, U1 snRNP recognizes the 5′ splice site through RNA base‐pairing [19] and helps suppress premature transcription termination (PTT) and intronic polyadenylation via interactions with RNA polymerase II [20, 21]. U2 snRNP, facilitated by SF3B, binds the branch point sequence (BPS) to modify exon inclusion levels [22, 23] and maintains splicing fidelity by stabilizing the catalytic core [19, 24] The RNA helicase Brr2 helicase activates the U4/U6/U5 tri‐snRNP [25, 26], and the U4/U6 assembly helps suppress spurious intronic polyadenylation [20, 27]. Meanwhile, the U6–U2 catalytic core aids in maintaining RNA integrity by preventing TUT1 from uridylation transcript [26, 28]. These results collectively demonstrate how snRNPs cooperate to preserve transcriptional surveillance [19, 24], and how their dysfunction leads to RNA integrity loss, underscoring the therapeutic potential of spliceosome targeting [20, 27].

**TABLE 1:** Roles of snRNPs in suppressing premature transcription termination and polyadenylation.

snRNP	Function in PTT and PCPA suppression	Mechanism
U1	Prevents PCPA at cryptic polyadenylation signals	U1 snRNA–pre‐mRNA base pairing, interaction with RNA polymerase II [19, 20]
U2	Ensures fidelity of splicing, prevents premature termination	Integration into spliceosome, facilitated by proteins like Prp [20, 29, 30]
U4	Suppresses global intronic PCPA events	Formation of U4/U6 di‐snRNP, tri‐snRNP assembly [27]
U6	Essential for splicing, prevents premature termination	Uridylylation by TUT1, formation of U4/U6 di‐snRNP [26, 28]

Abbreviations: PTT, premature transcription termination; PCPA, premature cleavage and polyadenylation.

Beyond the core snRNPs, additional factors contribute to the fine‐tuning of splicing. For example, the LUC7 protein family guides 5′ splice site selection via sequence‐specific binding, and its deficiency affects the splicing of metabolic genes in acute myeloid leukemia (AML) [31, 32]. The U1 snRNP‐associated factor ZNF207 affects cryptic splicing in the LMNA gene connected to progeria, while GEMIN5, vital for snRNP creation, is frequently mutated in neurodevelopmental disorders (NDDs) [33, 34]. Through RNA structural remodeling, RNA helicases such as Brr2 and DEAD‐box helicase 5 and 9 (DDX5 and DHX9) promote spliceosome activation and U2 recruitment [25, 35, 36].

In conclusion, the core spliceosome and its auxiliary partners constitute a multilayered regulatory system that guarantee precise splice site selection through RNA structural rearrangements, dynamic complex assembly, and cis‐element recognition. Dysregulation of this system results in splicing fidelity loss and is implicated in diseases including cancer and neurological disorders, underscoring the therapeutic relevance of spliceosome components.

### 2.2 Precise Recognition by Cis‐Elements and Trans‐Factors

Trans‐acting factors and cis‐regulatory elements interact dynamically to precisely regulate AS [37]. Core splice signals, such as the polypyrimidine tract, BPS, and 5′ and 3′ splice sites, as well as splicing enhancers and silencers, such as ESE, ISE, ESS, and ISS, optimize splice site usage and recognition [16, 38, 39, 40, 41]. Epigenetic context chromatin architecture and RNA secondary structures, such as G‐quadruplexes that obstruct splice motifs, all affect their function [16, 38, 42]; super‐enhancers can influence splicing via long‐range loops, as demonstrated by Klf5 regulation [43]. High‐throughput methods such as CRISPR–RfxCas13d now allow genome‐wide dissection of these elements [44, 45].

Trans‐acting factors, such as hnRNPs and SR proteins, decipher cellular signals to control splicing outcomes. Phosphorylation regulates SR proteins, such asSRSF1–3, which bind enhancers via RRM domains [46]. For instance, SRSF1 promotes CD44 and PTK2 isoforms [47, 48, 49] and activates NF‐κB signaling through ENPP3 splicing [50]. Cues like PTMs are integrate by hnRNPs: hnRNP L phosphorylation modulates the splicing of transcripts linked to apoptosis [51], and hnRNPC can identify m⁶A marks to affect splicing choices [52]. Phase separation of a number of factors, such as hnRNP A1 [53, 54, 55] and SRSF9 [56], result in the formation of regulatory condensates (Figure 1B). Splicing programs are further given contextual specificity by tissue‐specific regulators like ESRP1 [57, 58].

Splicing control is further diversified by RNA modifications, such as m⁵C deposited by Nsun2 [59] and atypical RNA‐binding proteins (RBPs) like QKI and CELF4 [60]. Splicing regulation is crucial in all biological contexts for the disruption of these processes results in aberrant splicing with pathological repercussions.

### 2.3 Dynamic Regulation by RNA Structure

By exposing or sterically blocking regulatory elements, RNA structural dynamics regulate AS and impact splice site accessibility and spliceosome assembly. Regulatory structures include Stem‐loops, G‐quadruplexes, long‐range interactions, and RBP‐mediated structural rearrangements, forming a responsive layer that integrates cellular signals [61, 62, 63, 64, 65, 66, 67] (Figure 1C).

RNA secondary structures play a crucial role in regulating splicing efficiency [40, 61]. Stable intramolecular structures are widespread and fine‐tuning splicing, as observed in yeast [68], while specific structures in human snRNPs provide platforms for splice factors [69]. G‐quadruplexes, including noncanonical variants, serve as molecular switches that regulate splice site accessibility via reversible folding [70, 71, 72]. For instance, the *TERT* gene's polymorphic G4 structures alter isoform ratios and affect cancer susceptibility [73, 74]. The dynamics of these structures are modulated by kinase signaling and helicases such as DHX36 and FANCJ, linking DNA damage response to RNA processing [75, 76, 77].

Splicing is also governed by long‐range RNA interactions and global conformational dynamics. Conformational sampling illustrates how RNAs transition between structural states to alter function [78, 79]. For instance, by stabilizing one of two conformations, bistable switches allow mutually exclusive exon selection [14, 15, 74, 80]. Inverted repeats or U‐rich elements can mediate long‐range base‐pairing, which can expose cryptic sites or promote back‐splicing and circRNA biogenesis [81, 82]. Exact rearrangements, such as large‐angle rotations in Group II introns during catalysis, have been discovered by structural biology [83, 84], and engineered systems like CIRC use self‐splicing introns for produce circRNA effectively [85]. Furthermore, ribonucleoprotein complexes like NF45–NF90 aid to splicing regulation by stabilizing duplex structures [86] and polypyrimidine tract‐binding protein 1 (PTBP1) dimers can trigger RNA looping to specify exon skipping or inclusion [87]. Viral RNAs utilize structural motifs for environment‐responsive circularization [88].

RBPs are pivotal in guiding structural transitions. They unwind local structures, stabilize functional conformations, or recognize specific architectural motifs, SF3B1 binding to adenosine stacks [67], for example. Proteins like DRB3D1 select flexible RNA substrates via conformational sampling [65], while signaling pathways like EGF affect splicing through RBP‐mediated structural alterations [64, 89].

Although it is still difficult to observe conformational changes in real time, developments in cryo‐EM, deep learning, and live‐cell imaging are transforming the study of RNA dynamics [61, 62, 63], Future interdisciplinary research will be essential to decode the principles of RNA structural regulation across the transcriptome. To sum up, through conformational switching, long‐range interactions, and RBP binding. RNA structure acts as a dynamic and controllable framework that crucially directs AS

### 2.4 Phase Separation in Splicing Regulation

LLPS facilitates the spatial arrangement of pre‐mRNA splicing in membraneless nuclear condensates like nuclear speckles [90, 91]. LLPS improves the efficiency and specificity of spliceosome assembly and splice site selection by concentrating splicing factors and pre‐mRNA substrates [92, 93]. Important splicing regulators, including SR proteins [56, 94, 95], hnRNPs [96], and other RBPs [97, 98], contain intrinsically disordered regions (IDRs) or prion‐like domains (PrLDs) that promote multivalent interactions driving condensate formation [99, 100]. Posttranslational modifications affect the dynamics of these assemblies. For instance, phosphorylation enhances the RNA binding of SRSF1 and promotes early spliceosome activation [101], whereas widespread phosphorylation of SRRM2 influences condensate characteristics and splicing results [102]. RNA modifications such as m⁶A also contribute by functioning as scaffolds or allosteric regulators [94, 95, 103, 104]. Additionally, condensate dynamics are influenced by metal ions including Zn^2^⁺ through proteins like TIA‐1 [105]. According to a recent study, ZFP207 undergoes LLPS with U1 snRNP via its C‐terminal IDR, which facilitating early spliceosome assembly and AS [92].

Biomolecular condensates serve as specialized reaction hubs that enrich splicing components, including U snRNPs and SR proteins, to optimize splicing efficiency and accuracy. They also act as scaffolds that direct AS decisions for specific transcripts [102, 106, 107]. For instance, WW domain containing adaptor with coiled‐coil (WAC) controls the splicing of genes linked to mitophagy, such as PINK1, by recruits RNA‐binding motif 12 (RBM12) into nuclear speckles via LLPS [108]. The lncRNA MALAT1 facilitates condensate formation with PTBP1 and hnRNPK to promote exon skipping [103]. OsU2AF35a in plants produces stress‐induced condensates that improve thermotolerance and allow for correct splicing of OsHSA32 pre‐mRNA [109].

LLPS interacts with cellular signaling pathways to facilitate the regulation of dynamic splicing. Kinases including PTK6 modify the phase separation of HNRNPH1 to alter splicing patterns [96], while acetyltransferases like NAT10 acetylate SRSF2 within condensates, affecting splice site selection [99]. Alternative exon inclusion or skipping depends on the stoichiometric balance and competitive dynamics between splicing factors with antagonistic functions, such as SRSF1 and hnRNP A1, within condensates; their dysregulation is associated with pathological splicing [110].

**FIGURE 2:**
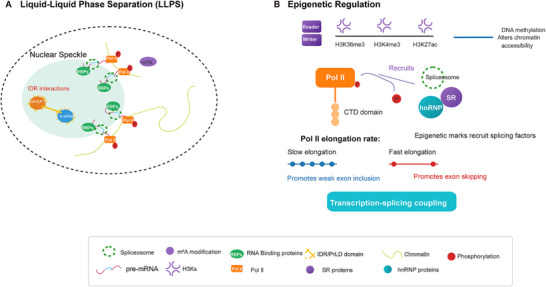
Higher‐order RNA structures and liquid–liquid phase separation (LLPS) in splicing regulation. (A) Biomolecular condensates formed via LLPS (e.g., nuclear speckles) serve as hubs for splicing regulation. Splicing factors (e.g., SRSF1, hnRNP A1) undergo multivalent interactions through their intrinsically disordered regions (IDRs) or prion‐like domains (PrLDs), concentrating snRNPs and substrate RNAs to enhance spliceosome assembly and regulatory specificity. (B) Epigenetic regulation (e.g., H3K36me3 marks) influences alternative splicing by modulating RNA polymerase II (Pol II) elongation rates and recruiting splicing regulators, thereby coupling transcription with splicing.

Dysregulation of LLPS represents an important mechanism underlying splicing defects in disease. Normal splicing patterns are disrupted by aberrant phase transitions, through gain‐of‐function (e.g., oncogenic condensates formed by SRSF9 [56], NAT10 [99], AKAP95 [111]) or loss‐of‐function (e.g., ATX [112], RBM20 mutations [113, 114], TDP‐43 aggregation [115]). Such disturbances are observed in a variety of diseases, including neurodegeneration, cancer, developmental disorders, and viral infection, underscoring the wide‐ranging physiological importance of regulated biomolecular condensation (Table 2 and Figure 2A).

**TABLE 2:** Phase separation mechanisms in alternative splicing regulation.

Factors	Target gene/splicing event	Disease	Key mechanism/interaction	References
SRSF2	BRCA1 (exon skipping)	AML	Phosphorylated SRSF2 enriches U1/U2AF via condensates; SRSF2^P95H^ disrupts RNA binding specificity	[95, 116]
SRSF9	SLC37A4 (alternative splicing)	Oral squamous cell carcinoma	Oncogenic LLPS drives pathogenic isoform selection	[56]
SRSF1 & hnRNP A1	Global exon–intron boundaries	*gastric cancer*	Antagonistic phase separation regulates exon inclusion vs. skipping	[110]
hnRNP A1	PKM (exon 9 skipping)	Multiple cancers	Condensates sequester weak 3′SS to promote exon exclusion	[117]
MALAT1‐PTBP1	NUMB (exon 12 skipping)	Lung cancer	Scaffolds PTBP1/hnRNP K into condensates repressing tumor‐suppressor exons	[103]
NAT10	YTHDF1 (exon skipping)	Gastric cancer	Acetylates SRSF2 via phase separation to drive oncogenic splicing	[99]
TDP‐43	STMN2 (neuronal splicing)	ALS, frontotemporal dementia	Aberrant phase separation yields insoluble aggregates disrupting RNA binding	[115]
RBM20	TTN, CAMK2D (exon skipping)	DCM	LLPS assembles spliceosome components; S637A mutant mislocalizes	[113, 114]
WASP	Cotranscriptional splicing	Wiskott–Aldrich syndrome	Forms condensates with SRSF2/Pol II; deficiency alters condensate properties	[118]
AKAP95	CCNA2, MYC (pre‐mRNA processing)	AML, solid tumors	Tyr‐dependent LLPS promotes oncogene splicing; YF mutation impairs mobility	[111]
USP42	Speckle‐associated mRNAs	NSCLC	DUB scaffolds PLRG1 into speckles via charge‐driven LLPS.	[107]
RBM10	NUMB (apoptosis genes)	Colon cancer, TARP syndrome	V354M mutation impairs condensate dynamics, causing exon skipping	[119]
WAC	Mitophagy genes (e.g., PINK1)	Cancer, neurodegeneration	Recruits RBM12 to regulate exon selection	[108]
SR30	Plant defense genes (exon retention)	Plant pathogen susceptibility	Nuclear condensates block splicing factor recruitment	[120]
OsU2AF35a	OsHSA32 (heat stress splicing)	Enhanced thermotolerance	Forms stress‐induced condensates via IDR; promotes proper splicing under heat stress	[109]
TaHRC	TaSR45a‐mediated splicing	Fusarium head blight resistance	LLPS modulates splice factor condensation; allelic variation affects disease susceptibility	[121]
EBNA1	SRRM1 (alternative splicing)	Nasopharyngeal malignancy	PrLD‐driven LLPS forms viral factories; interacts with SRSF1 to promote oncogenic splicing	[122]
FZP‐NAL1	Plant developmental genes	Plant developmental defects	NAL1 hexamer “molecular cage” modulates FZP phase separation extent	[123]
FBL	pre‐rRNA; oncogenes (e.g., MYC)	AML	Drives efficient pre‐rRNA processing, indirectly altering mRNA splicing balance	[124]

Abbreviations: AKAP95, A‐kinase anchoring protein 95; ALS, amyotrophic lateral sclerosis; AML, acute myeloid leukemia; CAMK2D, calcium/calmodulin‐dependent protein kinase II delta; CCNA2, cyclin A2; DCM, dilated cardiomyopathy; DUB, deubiquitinating enzyme; EBNA1, Epstein–Barr nuclear antigen 1; FBL, fibrillarin; FZP, FRIZZLE PANICLE; hnRNP A1, heterogeneous nuclear ribonucleoprotein A1; IDR, intrinsically disordered region; LLPS, liquid–liquid phase separation; MALAT1, metastasis‐associated lung adenocarcinoma transcript 1; MYC, MYC proto‐oncogene; NAL1, narrow leaf 1; NAT10, N‐acetyltransferase 10; NUMB, NUMB endocytic adaptor protein; NSCLC, non‐small cell lung cancer; OsHSA32, Oryza sativa heat stress‐associated 32; OsU2AF35a, Oryza sativa U2 auxiliary factor 35a; PINK1, PTEN‐induced kinase 1; PKM, pyruvate kinase M1/M2; PLRG1, pleiotropic regulator 1; Pol II, RNA polymerase II; pre‐rRNA, precursor ribosomal RNA; PrLD, prion‐like domain; PTBP1, polypyrimidine tract‐binding protein 1; RBM10/20: RNA‐binding motif protein 10/20; SLC37A4, solute carrier family 37 member 4; SR30, serine/arginine‐rich protein 30; SRRM1, serine/arginine repetitive matrix 1; SRSF1/2/9, serine/arginine‐rich splicing factor 1/2/9; STMN2, stathmin 2; TaHRC, Triticum aestivum histone‐related chromatin protein; TaSR45a, Triticum aestivum serine/arginine‐rich splicing factor 45a; TARP, talipes‐arthrogryposis‐renal defects‐pericarditis syndrome; TDP‐43, TAR DNA‐binding protein 43; TTN, titin; U1/U2AF, U1 small nuclear ribonucleoprotein/U2 auxiliary factor; USP42, ubiquitin‐specific peptidase 42; WAC, WW domain containing adaptor with coiled‐coil; WASP, Wiskott–Aldrich syndrome protein; YTHDF1, YTH N6‐methyladenosine RNA‐binding protein F1.

In summary, LLPS provides a flexible mechanism for orchestrating splicing regulation, which is further influenced by epigenetic modifications, a topic we will explore in the next section.

### 2.5 Epigenetic and Cotranscriptional Regulation

AS is well regulated by epigenetic mechanisms, which are part of a multilayered, cotranscriptionally based system of gene expression control [125]. Histone modifications, DNA methylation, and chromatin architecture collectively influence splice site selection by modulating RNA polymerase II elongation kinetics, recruiting splicing factors, or altering the local chromatin environment (Figure 2B). This integration enables precise spatiotemporal regulation of splicing outcomes [126, 127, 128, 129] and significantly enhances the efficiency and flexibility of gene expression [59, 125, 130].

Histone modifications are a crucial mechanism in AS. For instance, in *Arabidopsis* H3K36me3 readers like MRG2 enlist splicing factors to establish a direct connection between histone marks and clock genes AS [126]. SETD1B‐mediated H3K4me3 deposition ensures proper coordination of transcription and splicing during germ cell development in mouse spermatogenesis [127]. H3K27ac‐marked super‐enhancers support circRNA biogenesis in addition to enhancing transcription, highlighting the multipurpose function of histone modifications in integrating transcription with RNA processing [131]. On the other hand, AS of the histone variant macroH2A1 results in isoforms that modulate the repair of DNA damage, indicating a feedback loop in which splicing affects epigenetic regulation [132]. In esophageal cancer, Bclaf1 facilitates multilevel gene control by transcriptionally activating POLR2A and coregulates splicing through the cofactor SNRPA [129]. Conversely, spliceosome elements like U1 snRNP exhibit bidirectional crosstalk by suppress PTT, preserving chromatin accessibility and influencing Pol II processivity [19].

The spatial framework for splicing regulation is provides by nuclear architecture. Frequently found close to heterochromatin boundaries, transcriptionally active regions promote dynamic chromatin transitions that aid in cotranscriptional splicing [133]. Partially spliced transcripts are retained and matured by nuclear speckles, which serve as quality control centers [134]. Moreover, RNA modifications like m⁶A and RBPs like TDP‐43 add layers to cotranscriptional control by promoting spliceosome assembly and activity through phase separation or modification‐dependent recruitment [135].

DNA methylation modulates splicing both directly and through epigenetic crosstalk. RNA m⁵C modification regulates splicing of developmental genes via the Nsun2–Jarid2–Alyref axis, which influences histone modifications and chromatin accessibility [59]. Similarly, by inhibiting splicing factor activity, m⁶A modifications can modify splice site selection [94]. Epigenetic‐splicing interplay also contributes to broader regulatory programs such as cellular senescence, where DNA methylation changes accompany splicing reprogramming [136].

Neural biological processes, such as aging, stem cell maintenance, and neural development, are impacted by this integrated regulatory layer. For instance, SRSF10 regulates the splicing of targets like Myo5a during myelination, which in turn controls oligodendrocyte differentiation [137]. Epigenetic suppressors like REST, which stop erroneous gene expression, preserve stem cell identity [138]. Significant splicing changes occur with aging, as illustrated in Drosophila, where decreased Smu1 results in ovarian stem cells mis‐splicing [130].

There are still several important unanswered questions, including how histone marks are decoded into splicing instructions, how multifactor regulatory networks, which include transcription factors, splicing factors and epigenetic modifiers, are integrated, how noncoding RNAs function in feedback loops, and how to treat splicing defects [126, 131, 137]. These networks will be clarified, and therapeutic approaches will be advanced by future research that combines single‐cell multiomics, epigenetic editing, and targeted splicing modulation [139].

In conclusion, Pol II dynamics, chromatin state, and the recruitment of splicing regulators are all influenced by epigenetic and cotranscriptional mechanisms, which in turn finely regulate AS. Development and cellular function depend on this integrated control layer, and its dysregulation presents new targets for RNA‐directed and epigenetic treatments.

### 2.6 Environmental Cues Orchestrate Splicing Reprogramming

In response to environmental stresses like oxidative stress, hypoxia, and chemotherapy, cells quickly reprogram AS, allowing for adaptive modifications in gene expression that aid in survival and fate determination. Splicing factors serve as molecular hubs that mediate this reprogramming by converting stress signals into splicing alterations and creating a dynamic, context‐dependent regulatory network [60, 140]. Diverse stressors often converge on shared or distinct molecular pathways, eliciting wide‐ranging functional outcomes.

Stress‐induced changes frequently involve altered expression or activity of splicing regulators. For instance, some therapies increase the expression of QKI and CELF4, which encourage the biogenesis of circFLNB. This circular RNA can modulate Hippo signaling by acting as a competing endogenous RNA [60]. Conversely, SF3A3 enhance cell survival under stress by promoting the processing of a full‐length antiapoptotic isoform of FOS [141]. Hypoxia widely reshapes splicing profiles through pathways involving HIF‐1α. Under low oxygen, the eIF4F complex is recruited by a structured RNA element in the 5′UTR of β‐catenin mRNA, which improves translation and sustaining of Wnt signaling [142]. Context‐dependent splicing responses are triggered by oxidative stress. A mitochondria‐targeted uromodulin isoform that increases NAD+ and ATP synthesis in certain conditions is produced by stress‐induced splicing, aiding in cellular recovery [143, 144]. Others show how cell type and environment influence functional outcomes by modulating antioxidant response genes via ADAR1‐mediated RNA editing through signaling pathways like PI3K/Akt–HO‐1 [145].

Splicing factors serve as the primary integrators that convert stress signals into altered splicing of important target genes. Examples that demonstrate how splicing regulators link environmental cues to phenotypic adaptation include PTBP3‐mediated exon skipping in transcripts involved in copper metabolism [146] and Eftud2‐promoted skipping events that activate proliferative signaling [147].

Noncoding RNAs, including MALAT1 [148], circFLNB [60], circSRPK1 [149], HCG20 [140], and miRNAs [150, 151], further refine stress‐responsive splicing and impact metabolic, proliferation, and stress resilience processes.

In conclusion, splicing factors, signaling pathways, and noncoding RNAs are all involved in the multilayered mechanisms by which environmental stressors reshape splicing programs. Advanced transcriptomic and bioinformatic techniques will be needed for systematic decoding of these intricate networks to progress from descriptive observation toward mechanistic and predictive understanding.

### 2.7 Advances in High‐Throughput Splicing Analysis and Bioinformatics

Recent advances in high‐throughput technologies are transforming our comprehension of AS, providing unique insights into its roles in both health and disease. These tools aiding in identifying the intricate regulatory processes that underlie transcriptomic diversity in addition to improving the detection and measurement of splicing.

By directly capturing full‐length transcripts, long‐read sequencing platforms like Oxford Nanopore and PacBio overcoming the inherent drawbacks of short‐read approaches in reconstructing isoform architecture. This significantly improves the identification of circular RNAs, fusion transcripts, and novel isoforms. Together with ever‐evolving computational tools like IsoQuant, Bambu, and StringTie2, these technologies allow systematic and accurate transcriptome annotation, providing a robust foundation for mechanistic research [152, 153].

Single‐cell splicing omics resolves cell‐type‐specific splicing heterogeneity, which is frequently obscured in bulk analyses, by combining single‐cell RNA sequencing with specialized bioinformatics pipelines. Despite challenges from transcriptome sparsity, newly developed algorithms such as MARVEL [154], scASfind [155], and Shiba/scShiba [156] enable effective splicing quantification at the cell clusters level, shedding light on dynamic regulation throughout development and disease [157]. By preserving anatomical context and enabling the mapping of splicing patterns within tissue architectures, spatial transcriptomics further extends this resolution. The spatial dimension of splicing regulation is demonstrated by examples such as isoform switching of *Plp1* in the mouse olfactory bulb [158] and layer‐specific splicing changes during human cortical development [159].

Artificial intelligence and machine learning are increasingly applied to splice site prediction. Transformer‐based models can directly identify splice sites from long genomic sequences, outperforming conventional tools such as SpliceAI in accuracy. Large‐scale RNA‐seq datasets and clinical variant interpretations from resources like ClinVar [160, 161, 162] are used to validate these methods. By integrating several prediction tools, Integrative algorithms, such as Introme, enhance the evaluation of variant pathogenicity and facilitate the clinical application of genetic findings related to splicing. Together, these technologies expand our understanding of splicing regulation and help bridge the gap between basic research and clinical application.

Precise splicing control is achieved through the integration of multiple regulatory layers, such as the core spliceosome, cis‐ and trans‐regulatory elements, RNA structural dynamics, phase separation, and epigenetic modifications. Nearby splicing regulatory elements (SREs), like ESEs/ESSs, and their cognate RBPs, like SR and hnRNPs proteins, have an impact on the splicing outcome of an exon, but also do local RNA structures that alter site accessibility, like G‐quadruplexes. These processes can be compartmentalized within nuclear speckles formed via LLPS, which are regulated by RNA polymerase II elongation dynamics and histone marks, such as H3K36me3. In addition to ensuring splicing fidelity and adaptability, this extremely cooperative and dynamic network identifies disease‐related vulnerable nodes for therapeutic intervention.

### 2.8 Concluding Perspective

A multitiered regulatory framework for AS is described in this section. The fundamental structure is shaped by the dynamic interaction between spliceosome components, such as U1 snRNP that prevents premature termination, and cis‐regulatory elements like ESEs and ISSs. In structural modulation, conformational switches, which include RNA secondary structures like G‐quadruplexes and long‐range interactions, facilitate splice site exposure. The spatial organization achieved through phase separation permits splicing factors to form functional compartments within biomolecular condensates, such as nuclear speckles, aiding in regulatory specificity. Histone modifications and transcription‐coupled processes, such as RNA polymerase II kinetics, serve as upstream modulators of alternative splicing through epigenetic and cotranscriptional mechanisms. These multilayered regulatory mechanisms ensure splicing fidelity and plasticity, but also create numerous vulnerable nodes whose dysregulation, as discussed in the following sections, becomes a cornerstone of human pathogenesis.

## 3 Biological Functions of AS

AS serves as a key mechanism in eukaryotes gene regulation, dramatically expanding proteomic diversity and functional complexity by generating multiple mRNA variants isoforms [163, 164]. Beyond producing protein variants with distinct or opposing functions, AS also fine‐tunes gene expression via nonsense‐mediated mRNA decay (NMD) [66, 165].

This section provides a comprehensive overview of the biological roles of AS. The content includes the regulation of cell fate decisions, such as proliferation, differentiation, apoptosis, and autophagy, and covers stem cell pluripotency and lineage commitment. This section details the coupling of AS with NMD in gene regulation, underscoring its importance in aging, and its contributions to tissue development and organ specialization including nervous, cardiovascular systems. The section also details how AS mediates metabolic adaptation, stress responses, immune regulation, and controls protein subcellular localization.

### 3.1 Regulation of Cell Fate and Homeostasis

By regulating the expression of protein isoforms, AS serves as a central mechanism for maintaining tissue homeostasis. Through the generation of functionally distinct variants, AS enables dynamic and context‐specific control of cellular identity and function.

#### 3.1.1 Cell Proliferation and Differentiation

By controlling important factors in a spatiotemporally manner, AS acts as a molecular switch that guides cell fate. Splicing alterations during differentiation have a direct impact on cell identity by altering transcription factors and signaling molecules. In skeletal muscle development, two antagonistic isoforms are produced by AS of Fxr1 exon 15: Fxr1E15^−^ supports myoblast proliferation, while Fxr1E15⁺ promotes terminal differentiation and myotube formation [166]. In neurogenesis, downregulation of PTBP1 induces a switch in DPF2 exon 7 splicing, substituting the pluripotency‐associated DPF2‐S isoform with the proneuronal DPF2‐L variant to drive neural differentiation [167]. The polycomb component SUZ12 generates two splice isoforms, SUZ12‐S and SUZ12‐L, which differentially regulate H3K27me3 deposition while collectively sustaining differentiation potential in embryonic stem cells [168]. The splicing factor SRSF10, which targets genes like Myo5a to ensure proper myelination, is also necessary for oligodendrocyte differentiation [137].

AS aids in regulating proliferation by balance quiescence and cell cycle progression. The RNA helicase DHX16 prevents aberrant p53 activation and hematopoietic stem cell exhaustion by suppressing intron retention in Emg1, thereby sustaining ribosome biogenesis [169]. In pancreatic β‐cells, the Sox9–SRSF5 axis regulates splicing of genes involved in insulin secretion, supporting glucose homeostasis [170]. During osteoblast differentiation, upregulation of a functional NIBAN2 isoform enhances RUNX2 activity to promote bone formation [171].

Dysregulation of splicing‐dependent proliferation and differentiation underpins multiple diseases. Through oncogenic isoform switching, AS promotes proliferation, invasion, and resistance in cancer, including glioblastoma [135], breast cancer [172], medulloblastoma [147], oral squamous cell carcinoma [56], and gallbladder cancer [173].

In summary, AS provides essential regulatory precision during development and tissue maintenance by fine‐tuning cell proliferation and differentiation through isoform‐specific functions.

#### 3.1.2 Apoptosis and Autophagy

By regulating core apoptosis and autophagy genes, AS plays a pivotal role in determining cell survival contributing significantly to tissue homeostasis. In apoptosis, AS fine‐tunes cell death pathways through functionally antagonistic isoforms. Splicing modulators, for instance, can induce proapoptotic MCL‐1 isoforms to encourage cell death under specific conditions [174]. Splicing mutations in WFS1 lead to aberrant transcripts that increase β‐cell apoptosis under stress in Wolfram syndrome. illustrating how AS defects directly affect apoptotic pathways [175].

In autophagy, AS influences the splicing balance of autophagy‐related genes to modulate pathway activity. For instance, DDX24 inhibits autophagy via NF‐κB/BECN1 signaling by suppressing the long isoform of IKBKG (IKBKG‐L) [176]. By regulating RBM12‐dependent BECN1splicing, WAC connects nuclear condensates to mitochondrial regulation, its loss promotes the accumulation of promitophagic BECN1‐S isoforms [108]. In neurodegeneration, mis‐splicing of MAPT increases 4R‐tau expression, leading to autophagy abnormalities and neuronal death [177].

Notably, the response to chemotherapy is also influenced by the regulation of apoptosis and autophagy by AS. A recurrent splice isoform of NT5C2 (NT5C2ex6a) enhances resistance to 6‐mercaptopurine in B‐cell acute lymphoblastic leukemia [178], Conversely, chemotherapy induced splicing reprogramming in colorectal cancer (CRC) suppresses tumor progression and increases circFLNB expression, indicating that treatment can reshape the AS landscape [60].

In conclusion, AS finely regulates the imbalance of apoptosis and autophagy genes, serving as a critical determinant of cell fate. Its precise control is essential for physiological homeostasis, and its dysregulation represents a key mechanism underlying disease pathogenesis and treatment resistance.

#### 3.1.3 Stem Cell Pluripotency and Lineage Commitment

AS is central to the maintenance of stem cell pluripotency and the guidance of lineage commitment, dynamically regulating the isoform balance of core transcription factors and chromatin‐modifying complexes throughout development [179].

During early embryogenesis, AS acts as a molecular switch governing the transition from totipotency to pluripotency. During maternal‐to‐zygotic transition zygotic splicing activation occurs and is dependent on phosphorylation‐mediated regulation of SF3B1 [180]. Correct splicing patterns are essential for embryonic development, as errors can lead to nonfunctional isoforms that disrupt lineage specification. The long nonoding RNA LincGET influences the initial cell fate decision in mouse embryos by inhibiting exon‐skipping splicing of CARM1, thereby modulating CARM1 protein levels [181].

In pluripotency maintenance, AS fine‐tunes gene expression programs by regulating functionally specialized isoforms of chromatin modifiers. The PRC2 component SUZ12 produces two splice isoforms with distinct roles: SUZ12‐S mediates H3K27me3 deposition at promoter‐proximal regions to repress target genes, while SUZ12‐L maintains global H3K27 methylation levels. Both are required for pluripotency and neural differentiation capacity in embryonic stem cells [168]. Similarly, PTBP1 regulates splicing of DPF2 exon 7 to determine BAF chromatin complex targeting: PTBP1 represses exon 7 inclusion in undifferentiated cells, producing DPF2‐S which binds pluripotency factor loci; upon neural differentiation, PTBP1 downregulation leads to exon 7 inclusion and DPF2‐L production, redirecting BAF to neurodevelopmental genes [167].

By modulating splicing patterns of fate‐determining factors, AS promotes tissue‐specific differentiation during lineage commitment. PTBP2 regulates splicing of targets like SYNGAP1 to affect neurogenesis in neural development [182]. Functionally opposing isoforms are produce by AS of FXR1 exon 15 in myogenesis: Fxr1E15^−^ maintains myoblast proliferation, while Fxr1E15⁺ promotes myotube differentiation and fusion [166].

In summary, AS establishes a complex regulatory system that promotes stem cell pluripotency and guides lineage commitment by precisely control of chromatin modifiers, transcription factors, and signaling pathways, providing essential insights into cell fate determination.

### 3.2 Gene Regulation via NMD

AS couples with NMD to dynamically control gene expression through the generation of transcripts containing premature termination codons (PTCs) [165]. This AS–NMD axis serves as a critical posttranscriptional regulatory mechanism that fine‐tunes proteome diversity and contributes to cellular homeostasis, differentiation, and stress adaptation.

Degrading PTC‐containing transcripts is a crucial role of unproductive splicing, which preventing the accumulation of truncated or harmful proteins. NMD proceeds via exon junction complex (EJC)‐dependent decay, triggered when a stop codon precedes the final splicing‐deposited EJC [183], or via EJC‐independent decay mediated by long 3′ UTRs, sequence motifs, or RNA structures [184]. With approximately 15% of human transcripts being NMD targets, this process is widespread role in adaptive gene regulation and RNA quality control.

Cellular homeostasis is maintained in large part by AS–NMD pathway. It promotes neuronal integrity and reduces harmful protein accumulation in neurons [185]. Additionally, it also buffers transcriptional noise to stabilize mRNA expression through a process termed gene‐specific transcriptional buffering [186]. Furthermore, NMD activity is highly responsive to developmental and environmental cues. During neural differentiation, NMD sensitivity is reprogrammed across hundreds of transcripts, directly shaping cell fate decisions [187]. In plants, cold stress engages AS–NMD, with CBF transcription factors enhance the splicing efficiency of cold‐responsive genes to promoting adaptation [188].

Splicing factors frequently autoregulate their expression via AS–NMD feedback loops, ensuring precise control of spliceosomal components and genomic stability. SF3B3, for example, harbors a structured region that suppresses a poison exon; when this structure is destabilized, exon inclusion introduces a PTC and triggering NMD of SF3B3 mRNA [189]. Similar autoregulatory circuits operate across numerous splicing factors, underscoring the evolutionary conservation and functional significance of this regulatory strategy.

In summary, the coupling of AS with NMD provides a versatile and dynamic system for fine‐tuning gene expression. Through unproductive splicing, autoregulatory feedback, and context‐dependent decay, this axis maintains proteomic balance and supports adaptive responses across developmental and environmental cues.

### 3.3 Aging

Aging is characterized by functional decline and increased disease susceptibility, underpinned by genomic instability, telomere shortening, and loss of proteostasis [4]. AS is a central player in this process, not merely a biomarker but an active driver that shapes transcriptomic and proteomic states [190]. Aged tissues exhibit broad AS dysregulation, including decreased splicing fidelity, elevated intron retention, and altered exon inclusion, collectively promoting cellular senescence and tissue dysfunction [12].

Age related loss of splicing factors is a major driver of AS dysregulation. Reduced expression or activity of core spliceosomal components such as U2AF1, PRPF19, and YBX1 leads to mis‐splicing of genes essential for tissue homeostasis. In bone marrow stromal cells, for instance, YBX1 deficiency disrupts osteogenic splicing programs and accelerates osteoporosis [191].

Tissue‐specific AS contribute to various age‐related pathologies. In the brain, mis‐splicing of synaptic genes such as CPNE1 and loss of regulators like hnRNP D‐like and TDP‐43 contribute to cognitive decline and AD [192]. In skeletal muscle, aberrant splicing of contractile genes promotes sarcopenia [193], while in the heart, age‐dependent accumulation of the MRG15L splice variant impairs regeneration [194]. Senescence‐associated autophagy can further redirect splicing toward proinflammatory translation [195].

Targeting AS dysregulation offers a promising strategy for healthy aging. Compounds such as doxifluridine can reverse AS defects and extend lifespan in C. elegans via modulating bacterial metabolism and splicing factor activity [196]. Small molecules like sciadopitysin stabilize splicing factors and ameliorate age‐related conditions such as osteoporosis in mice [191], while MEK inhibitors reduce senescent cells burden and modify splicing profiles in progeria models [197]. These findings highlight the potential of splicing corrective therapies to promoting healthspan.

In summary, AS is both a marker and a driver of aging, with its dysregulation actively promoting cellular and organismal decline. Deciphering splicing networks in aging could inform strategies to extend healthspan and counter age‐related diseases.

### 3.4 Tissue Development and Organ Specialization

Tissue and organ specialization depends on highly specific AS programs that fine‐tune structural and signaling protein isoforms, ensuring precise physiological function across diverse systems.

#### 3.4.1 Nervous System Development and Function

AS is essential for nervous system development and functional, dynamically shaping neural gene expression to generate the proteomic diversity required for neuronal identity, synaptic plasticity, and circuit assembly.

During neural development, AS directs cell fate by modulating transcriptional and chromatin regulators. PTBP1 represses DPF2 exon 7 to maintain a pluripotency‐associated isoform, with its downregulation promotes the proneural DPF2‐L variant that drives differentiation [167]. SRSF10 is required for oligodendrocyte differentiation and myelination [137], while mutations in SNW1 impair neurogenesis and cause microcephaly, highlighting the essential role of AS in neural development [198].

In neuronal specification, AS drivers axon guidance and adhesion molecules to refining connectivity. In *C. elegans*, approximately 25% of neuronal genes, including unc‐40/DCC and sax‐3/ROBO, undergo AS, enhancing neuronal diversity and connection precision [45]. In mammals, teneurin‐3 isoforms with EGF repeats adopt compact conformations that promote synaptic specificity and neural circuit assembly [199].

In mature neurons, AS continues to regulate synaptic function and adaptability: PRMT9‐mediated splicing of synaptic genes including NRXN2 and SYN1 supports cognitive [200], while stress‐induced splicing reprogramming modulates neurotransmission and blood–brain barrier integrity [201].

In summary, AS shapes neural development, circuit assembly, and functional plasticity through precise control of gene isoforms, with its dysregulation underlying many neurological diseases.

#### 3.4.2 Cardiovascular System Homeostasis

AS is essential for cardiovascular development and function, regulating genes involved in heart morphogenesis, contractility, and electrical activity [202, 203, 204, 205]. During cardiogenesis, it guides cell lineage specification and structural formation: PTBP1 regulates Arrb1 splicing in endothelial cells, affecting migration and proliferation for ventricular development [203], while the lncRNA CARMEN produces isoforms that act as a lineage switch, balancing cardiomyocyte and smooth muscle differentiation [206].

In the adult heart, AS sustains contractile and electrophysiological function via sarcomeric and calcium handling genes. QKI regulates splicing of over 1000 targets to preserve sarcomere integrity [205], DDX5 prevents pathological CaMKIIδ isoforms to maintain calcium homeostasis [204], and RBPMS modulates titin splicing to ensure proper passive tension and diastolic function [207, 208].

#### 3.4.3 Lung Homeostasis

AS contributes to lung homeostasis by modulating metabolic, immunological, and structural processes. It regulates genes controlling cellular metabolism and energy utilization [209], preserves genomic integrity via regulators like MEN1 [210], supports autophagy through factors such as DDX24 [176], and shapes immune responses via variants of ATP11A and DPP9 [211]. Together, this mechanism fine‐tunes essential metabolic and immune functions for pulmonary health.

#### 3.4.4 Reproductive System Development

AS ensures fidelity in germ cell development and gametogenesis by precisely regulation meiotic and spermatogenic genes. The hnRNPC–HuR complex preserves correct splicing of meiotic genes such as *Sycp1* and Brca1 [52], while SRSF1 [212] and CWF19L2 [213] maintain splicing precision during spermatogenesis, controlling genes including *Stra8*, *Dazl*, *Znhit1*, and *Rbfox1* to support fertility. In summary, AS centrally governs tissue development and organ specialization by dynamically regulating differentiation, structural integrity, and functional adaptation, fine‐tuning gene expression programs essential for organismal homeostasis.

### 3.5 Mediating Stress Response and Metabolic Adaptation

AS serves as a critical mechanism for cellular and organismal adaptation to environmental stressors, including hypoxia, oxidative stress, and nutrient fluctuation. By rapidly modulating isoform profiles of metabolic enzymes, signaling molecules, and stress‐response factors, AS drives metabolic reprogramming and enhances fitness under dynamic conditions.

#### 3.5.1 Metabolic Adaptation through Splicing Reprogramming

AS supports metabolic plasticity by fine‐tuning the expression and activity of enzymes and regulators governing energy and lipid metabolism. In response to fluctuating nutrient levels or energy demands, cells reprogram splicing to optimize pathway flux and preserve homeostasis.

In energy metabolism, AS reshapes glycolytic output, for example, activated immune cells, repress of PKM1 to favor the PKM2 isoform, boosting glycolytic flux to sustain effector functions [214]. Under hypoxia, splicing‐derived isoforms promote mitochondrial resilience and maintain oxidative metabolism [143, 215].

Lipid metabolism is similarly regulated by AS, with SR proteins and other splicing factors generating lipogenic enzyme isoforms that tune lipid synthesis and storage. In hepatocytes, metabolic stress reprograms lipin‐1 splicing to rebalance lipid utilization [216], while in the lung, *PKM* splicing modulates glycolytic and biosynthetic flux in fibroblasts, influencing collagen production and tissue remodeling [209].

Mitochondrial function and cellular energy sensing are also modulated via AS. Exon choices in genes such as *Mff* regulate phosphorylation and mitochondrial dynamics [217], while nutrient‐sensing pathways including mTORC1 reprogram splicing factor activity to reshape metabolic isoforms across energy, nucleotide, and amino acid pathways [218]. Such AS‐driven metabolic adaptation has been linked to lifespan regulation [219].

#### 3.5.2 Roles of AS in Diverse Stress Responses

AS enables rapid proteome diversification under abiotic, chemical, and biotic stress. Hypoxia and oxidative stress induce isoforms that enhance mitochondrial function and promote survival [143], while tumors exploit HIF‐1α and SRSF6‐dependent splicing programs to balance apoptosis and proliferation, with SRSF6 levels correlating with prognosis [220, 221]. AS also contributes to evolutionary and immune adaptation, for example, an NFKB1 splice variant dampens immune activation in high‐altitude Andean populations [222], and NF‐κB isoforms fine‐tune inflammatory responses [223]. Psychological stress further reconfigures neural splicing programs to modulate neurotransmission and barrier integrity, highlighting AS as a coordinator of systemic stress responses [201].

Chemical and pharmacological stressors also engage splicing networks. Various therapeutics influence splicing factor activity and shift isoform expression of genes controlling signalling and metabolism [224, 225]. Chemotherapy can select for splice variants that enhance stress tolerance, contributing to adaptive resistance [178]. Spliceosome dysfunction itself triggers JNK/p53 signaling, cell cycle arrest, and apoptosis, revealing intrinsic surveillance mechanisms that monitor splicing fidelity [226].

Biotic stress similarly relies on AS for immune regulation. Bacterial infection induces splicing factor dephosphorylation [227] and drives the production of immune‐modulatory transcripts [228], whereas plant immunity involves methylation‐dependent splicing of defense genes [229]. Viral infections provoke widespread host splicing changes that shape interferon signaling and antiviral responses [217]; and RBM45 ensures proper splicing of antiviral genes to restrict infection [230, 231].

Together, these context‐dependent splicing programs orchestrate metabolic adaptation and stress resilience across tissues and species, underpinning physiological homeostasis in fluctuating environments.

### 3.6 Regulation of Immune Responses

AS serves as a central mechanism for the regulation of immune function, dynamically modulating the expression of immune‐related genes to effector responses, cell development, activation, and homeostasis. This makes it possible to precisely and flexibly regulate both innate and adaptive immunity.

#### 3.6.1 Innate Immune Regulation

AS is essential for optimizing innate immune signaling pathways. A primate‐specific IFNAR2 splice variant moderating type I interferon signaling to prevent excessive inflammation during viral infection by acting as a decoy receptor in the interferon response [232]. Mitofusin Mff splicing modifies antiviral responses by altering exon inclusion, which impacts downstream MAVS pathway activity and AMPK phosphorylation [217]. Additionally, AS of IL1RN can produce anti‐inflammatory isoforms that reduce myeloid inflammation, demonstrating the regulatory checkpoint function of AS in immune activation [233].

#### 3.6.2 Adaptive Immunity and Tumor Immune Evasion

AS influences immune recognition and response by regulating T cell activity and antigen expression. Splicing‐derived PD‐1 variants modulate immune activity by inhibiting CD8⁺ T cell proliferation and effector functions during T cell activation [234, 235]. Immune recognition may be impacted by altered splicing of surface antigens like CD20, which can lower target expression [236]. Similarly, inactive isoforms that avoid immune‐mediated cell death [237] may result from tumor‐specific splicing of genes like GSDMB. Additionally, IL‐18 splicing can result in shortened variants that affect T cell suppression and regulation [238].

#### 3.6.3 Immune Cell Lineage Specification

AS guides immune cell fate and function by regulating key transcriptional and metabolic pathways. In T helper cells, splicing of factors like T‐bet reinforces Th1 identity [239], while isoform switches in metabolic enzymes such as PKM enhance glycolysis in CD8⁺ T cells, supporting effector responses [214].

#### 3.6.4 Regulation in Autoimmunity and Inflammation

AS is involved in the regulation of inflammatory reactions and immune tolerance. Immune genes have been found to have cell‐type‐specific and population‐associated splicing variations, including splicing quantitative trait loci (sQTLs), some of which are correlated with autoimmune susceptibility [240]. As decoy receptors, truncated splice variants of receptors like IL13RA1 alter cytokine signaling and affect allergic and antiparasitic reactions [241]. AS plays a protective role in inflammatory contexts because stress‐induced isoform switching, as seen in genes like uromodulin and CEACAM1, improves tissue resilience and reduces inflammatory damage [143, 220].

#### 3.6.5 Evolutionary and Species‐Specific Adaptations

AS events unique to a species support environmental fitness and immune adaptation. Differences in stress tolerance in plants are caused by allelic variation in splicing factors like OsRS2Z38, which permits effective splicing of cold‐responsive genes in adapted varieties [242]. An isoform that controls seed dormancy and drought response is produced by triticeae‐specific splicing in TaPP2C‐a5, providing an adaptive advantage in arid environments [243]. Additionally, plant circRNAs produced by back‐splicing enhance regulatory networks via RBP binding or miRNA sequestration, demonstrating the functional diversification made possible by AS across species [60].

In conclusion, AS allows for precise control over innate and adaptive responses, immune cell identity, and inflammatory balance by dynamically shaping the immune transcriptome. Its essential function in immune homeostasis is highlighted by its contextual regulation and evolutionary adaptability.

### 3.7 Regulation of Subcellular Localization

AS precisely controls protein subcellular localization by modulating targeting signals, domain composition, and interaction interfaces. This posttranscriptional regulation enables functional specialization, implicated processes such as differentiation, stress adaptation, and signal transduction [7].

#### 3.7.1 Regulation of Nucleocytoplasmic Shuttling

AS directs nuclear‐cytoplasmic proteins trafficking by including or excluding exons encoding nuclear localization (NLS) or nuclear export signals motifs. During osteoblast differentiation, a NIBAN2‐derived isoform promotes a RUNX2 variant with an intact NLS, enhancing nuclear import and osteogenic genes activation [171]. TDP‐43 facilitates ALKBH5 nuclear import, producing the oncogenic CDC25A‐1 splice variant and driving tumor proliferation [135]. Hypoxia‐induced DDX3X cleavage generates a nuclear fragment that reprograms splicing for metabolic adaptation [215], while ionizing radiation induces nuclear colocalization of splicing regulators like TXNL4B to modulate DNA repair and cellular radioresistance [244].

#### 3.7.2 Organelle Targeting and Membrane Association

AS also directs protein targeting to specific organelles and membranes by altering domain architecture or membrane‐anchoring motifs. A hypoxia‐induced uromodulin isoform is rerouted to mitochondria, where it supports metabolic protection and energy production [143]. The adaptor protein WAC uses its WW domain to localize to nuclear speckles and recruits RBM12, thereby controlling the splicing of mitophagy‐related genes [108]. Isoforms diversity further shapes membrane splicing: KRAS4A and KRAS4B differ in their C‐terminal sequences, conferring distinct membrane affinities and signaling outputs [245]. In neuronal, alternative translation start sites in NPR receptors generate both transmembrane and soluble isoforms, expanding their functions in synaptic organization and extracellular signaling [246]. Loss of RBFOX2 induces an ABI1ΔEx9 isoform that relocates from the cytoplasm to the leading edge of migrating cells, promoting invasion and metastasis [247].

#### 3.7.3 Reprogramming Chromatin Complex Localization

AS can redirect chromatin‐modifying complexes to specific genomic loci, thereby shaping transcriptional programs and cell fate decisions trajectories. During neural differentiation, PTBP1‐mediated splicing of *DPF2* generates the DPF2‐S and DPF2‐L isoforms, which exhibit distinct binding preferences for pluripotency enhancers and promoter regions, consequently modulating chromatin remodeling and differentiation [167]. Likewise, AS of *SUZ12*, a core PRC2 component, produces isoforms that differentially influence H3K27me3 deposition, providing a mechanism to fine‐tuning developmental gene expression [168].

#### 3.7.4 Localization and Functional Networks of Splicing Factors

The activity of splicing factors is itself tightly regulated by subcellular localization, posttranslational modifications, and their capacity to undergo phase separation [7]. For example, circPPAP2B promotes the nuclear translocation of HNRNPC to modulate splicing outcomes [248], whereas SMNDC1 relies on its Tudor domain for nuclear speckle localization, a process that is disrupted by small‐molecule inhibition [249]. Within these subnuclear compartments, splicing regulators such as PRPF40A, SRRM2, and SON assemble into functional modules that cooperatively regulate exon inclusion [40, 250, 251]. Posttranslational cues further tune their activity, exemplified by CDK11‐mediated phosphorylation of SF3B1 during embryonic development [180]. Additionally, several RBPs, including ADAR1, generate isoforms with distinct localizations: nuclear variants drive RNA editing, whereas cytoplasmic forms redistribute to stress granules under stress conditions [252].

In summary, AS dynamically controls protein localization, shaping functional specificity across spatial and temporal contexts. This mechanism underpins cellular adaptation and functional diversity, though real‐time, high‐resolution tracking [253] of splice variant localization in living systems remains technically challenging.

### 3.8 Concluding Perspective

AS enhances eukaryotic complexity by diversifying the proteome, fine‐tuning gene expression through mechanisms such as NMD and enabling rapid adaptation to environmental changes. It supports key physiological processes, including differentiation, organ development, immune regulation, and stress response, promoting organismal fitness.

However, the precision and versatility of splicing also make it vulnerable: dysregulation can disrupt gene networks and drive disease. Understanding the physiological roles of splicing thus provides crucial context for exploring its contributions to disorders and potential therapeutic strategies.

## 4 AS in Human Diseases

The central role of AS in maintaining cellular homeostasis, development, and stress responses implies that its dysregulation may result in pathological consequences [5, 8, 12, 254, 255]. Indeed, AS is not a passive bystander but an active disease driver rather than a passive bystander. As detailed below, by interfering with essential physiological functions, dysregulated AS plays a role in the pathophysiology of almost all significant human diseases.

**FIGURE 3:**
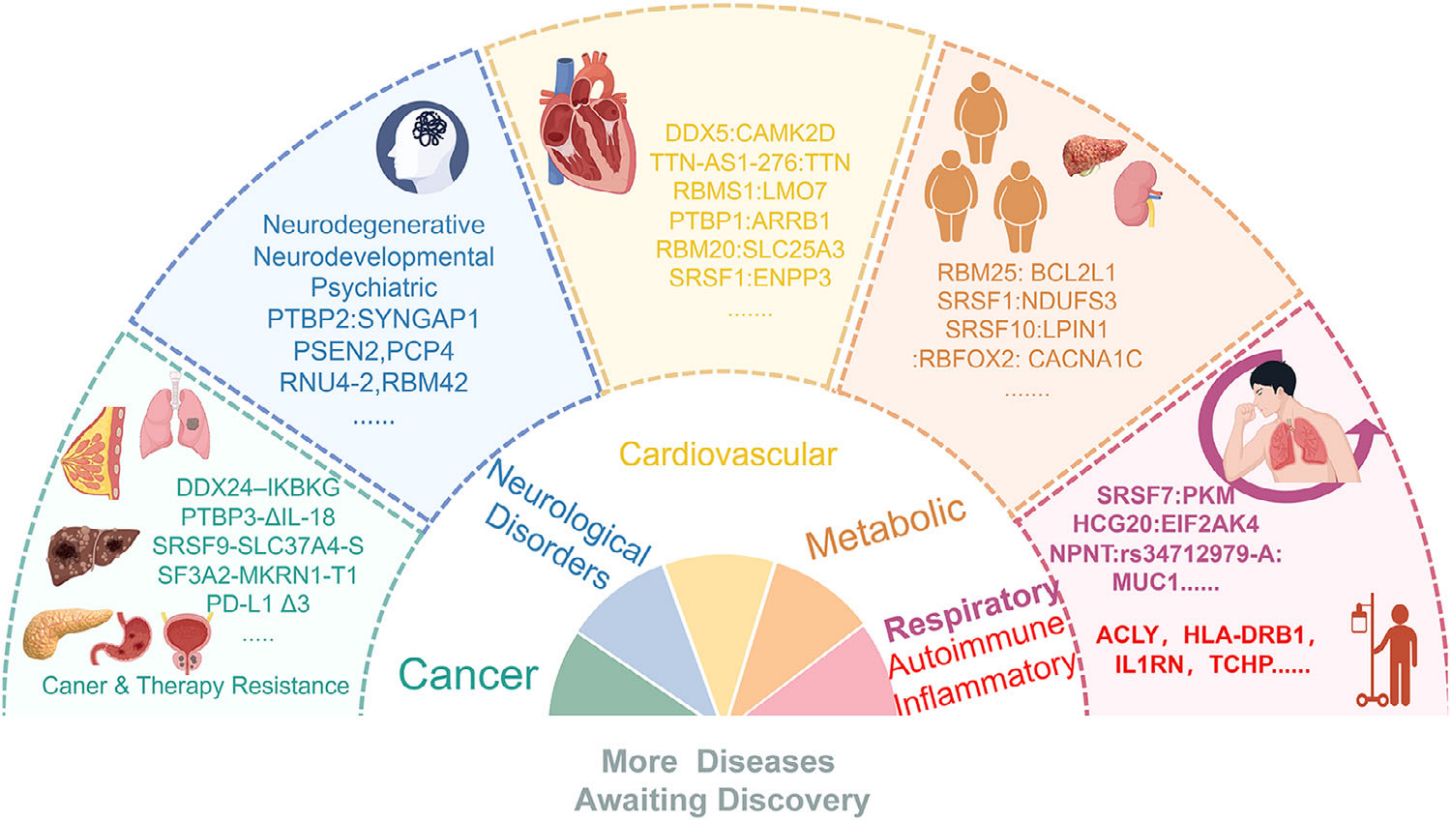
Role of alternative splicing in human diseases. Alternative splicing in cancer, neurological disorders, cardiovascular diseases, metabolic diseases, respiratory diseases, autoimmune inflammatory diseases, and other diseases. Specific alternative splicing sites associated with each disease are listed in the figure.

This section offers a methodical summary of how splicing dysregulation causes a wide range of human diseases, including cancer, including solid tumors, hematologic malignancies, and therapy resistance, neurological disorders, including neurodegenerative, neurodevelopmental, and psychiatric conditions, cardiovascular diseases, autoimmune and inflammatory disorders, metabolic syndromes, and respiratory ailments (Figure 3). It highlights aberrant splicing as a key cause of disease by emphasizing common pathogenic mechanisms such as mutations in core spliceosomal components, aberrant phase separation, and rewiring of cellular pathways.

### 4.1 Common Mechanisms of Splicing Dysregulation in Human Diseases

The dysregulation of AS is a hallmark of numerous human diseases, contributing to pathogenesis through the production of aberrant protein isoforms that disrupt cellular homeostasis. A remarkable convergence of molecular mechanisms underlies this dysregulation across disease spectrums, even though the phenotypic manifestations of splicing defects are diverse, ranging from cancer to neurological, cardiovascular, and metabolic disorders. These recurring pathogenic themes place aberrant splicing at the center of disease pathophysiology rather than as passive observers. Comprehending these shared mechanisms offers an effective conceptual framework for classifying illnesses and formulating comprehensive treatment approaches. In the following Sections 4.2–4.8, specific disease examples will be used to illustrate the five fundamental mechanisms that universally contribute to splicing‐related pathologies.

#### 4.1.1 Recurrent Mutation or Dysregulation of Core Spliceosomal Components

Mutations encoding core spliceosomal proteins and regulatory factors like SF3B1, SRSF2, and U2AF1 are hallmark drivers in hematological malignancies [256, 257]. Notably, solid tumors and neurological disorders often exhibit dysregulation of these same factors, though they are less frequently mutated. For instance, PTBP1 is upregulated in glioma to promote oncogenic splicing [258] but is downregulated during normal neuronal differentiation to allow for proneural splicing programs [167]. This implies that the precise expression level of core splicing factors is critical for cellular homeostasis across tissues.

#### 4.1.2 Aberrant Phase Separation and RNP Granule Pathogenesis

Dysregulation LLPS, outlined in Section 2.4, represents a unifying mechanism. It can manifest as gain‐of‐function that generate oncogenic condensates, exemplified by SRSF9 or hnRNP A1 in cancer [56, 120] (Section 4.2.1), or loss‐of‐function, leading to toxic aggregation or mislocalization as seen for TDP‐43 in ALS/FTD [115] and RBM20 [113, 114]. The specific consequences of LLPS dysregulation in cancer (Section 4.3) and neurodegeneration (Section 4.4) will be examined in the following sections.

#### 4.1.3 Splicing‐Mediated Rewiring of Core Cellular Pathways

Pathological AS frequently affects genes in highly conserved cellular pathways, resulting in their coordinated dysregulation and contributing to a range of disease phenotypes. For example, chemotherapy resistance [141] and metabolic diseases like Wolfram syndrome brought on by WFS1 mutations are caused by disruption of apoptosis and autophagy balance [175]. Similar to this, splicing functions as a metabolic switch in diseases like fatty liver disease through changed PKM2/PKM1 ratios reprogram glycolytic flux [209] where isoform changes in LIPIN1 and cancer [216].

By creating immunosuppressive isoforms, such as truncated ΔIL‐18 in gallbladder cancer, along with metabolic and survival pathways, tumors use AS to evade immune surveillance [238] (a topic detailed in Section 4.2). Collectively, these examples highlight how misregulated splicing can simultaneously perturb multiple critical cellular functions, amplifying disease progression and therapeutic resistance.

#### 4.1.4 Environmental Stress‐Induced Splicing Reprogramming

Rapid AS reprogramming as a primary response to stressors like hypoxia, oxidative stress, and chemotherapy [60, 143, 201]. Although this response is initially adaptive, it has the potential to become maladaptive and locked‐in, sustaining disease states like fibrosis, chemoresistance, and neuronal dysfunction. This mechanism emphasizes how the environment shapes splicing landscapes that are specific to diseases.

#### 4.1.5 Noncoding RNAs as Unifying Regulatory Hubs

CircRNAs and lncRNAs are increasingly recognized as master regulators of splicing factors across diverse pathological contexts, often exerting broad influence over splice‐site selection and transcriptome remodeling. In cancer, for instance, circSRPK1 amplifies oncogenic signaling by enhancing the activity of key splicing regulators, thereby promoting malignant isoform programs in gastric tumors [149]. Similarly, in pulmonary hypertension, the super‐enhancer‐driven lncRNA HCG20 stabilizes U2AF2 and enforces pathogenic splice‐site usage, illustrating how a single dysregulated noncoding RNA can hijack the splicing machinery to drive disease progression [140]. Together, these examples underscore a unifying paradigm in which noncoding RNAs function as upstream orchestrators of splicing dysregulation, shaping disease trajectories across seemingly disparate organ systems.

Building on this conceptual framework, the following sections synthesize how five overarching mechanisms, dysregulation of core spliceosomal components, aberrant phase separation, pathway rewiring, stress‐responsive splicing programs, and splicing modulation by noncoding RNAs, converge to produce disease‐specific phenotypes. This integrated perspective not only clarifies the molecular underpinnings of splicing‐driven pathogenesis but also highlights a compelling therapeutic opportunity: interventions that target these conserved regulatory mechanisms may offer broad, cross‐disease applicability and transformative potential.

### 4.2 Cancer

AS frequently exhibits a dual nature, producing either tumor‐suppressive variants that impede the progression of cancer or pro‐oncogenic isoforms that drive malignancy. It plays a complex and crucial role in tumorigenesis, progression, and therapy response [8, 9]. Tumor type, cellular context, and microenvironmental signals all play a major role in this duality [259]. Advances in high‐throughput sequencing and molecular biology have greatly expanded our understanding of splicing reprogramming in cancer, providing a foundation for novel diagnostic and therapeutic strategies. The dysregulation of AS has been most extensively characterized in cancer, serving as a paradigm for understanding its pathological mechanisms across human diseases.

#### 4.2.1 Solid Tumors

The pathogenesis of solid tumors exemplifies multiple common mechanisms of splicing dysregulation introduced in Section 4.1. These include mutational activation of splice factors (e.g., SF3B1 in uveal melanoma [260]), aberrant phase separation (e.g., SRSF9 condensates in OSCC [56]), and splicing‐mediated immune evasion (e.g., GSDMB isoforms [237]). Below we detail these mechanisms across different cancer types

In lung cancer, multiple layers of splicing dysregulation have been identified. For instance, DDX24 promotes tumor growth by modulating IKBKG splicing to suppress autophagy [176], whereas loss of MEN1 induces widespread splicing defects and genomic instability via disrupted transcription elongation [210]. Genetic variants such as the sQTL rs156697‐G alter GSTO2 splicing to promote proliferation [261], and lncRNAs like ITPR1–AS1 facilitate oncogenic HRAS splicing and metastasis [262]. ZEB1 enhances invasiveness by promoting CD44 AS and reshaping the tumor microenvironment [263]. Epigenetic silencing of the tumor‐suppressive PRSS3‐V3 isoform via intronic CpG methylation is associated with early disease progression [264].

Gastrointestinal cancers frequently exhibit splicing dysregulation. In CRC, SRSF1 promotes tumor progression by sustaining TIMP1 exon inclusion [265], whereas PTK6 induces autophagy via phase separation‐mediated splicing of NBR1 [96]. sQTLs such as rs61746794‐T increase expression of oncogenic PRMT7 isoforms [266]. Conversely, restoration of tumor‐suppressive isoforms like TIMP1Δ4 can inhibit metastasis [60]. In gastric cancer, circRNA‐mediated pathways drive expression of oncogenic *RON* variants [149], and PTBP3‐mediated skipping of COX11 exons facilitates resistance to cuproptosis [146]. Similar mechanisms operate in pancreatic [247], gallbladder [238], cholangiocarcinoma [267], oral cancer [56], esophageal cancer [129], and liver cancers [268], often involving dysregulation of splicing factors like RBFOX2 [247], HNRNPR [268], and NONO [173].

In breast cancer, AS events contribute to subtype‐specific pathogenesis. For example, the CCND1b isoform accelerates cell cycle progression via CDK4/CyclinD1–pRB–E2F1 signaling and correlates with poor prognosis [269]. In TNBC, MYC enhances U2SURP translation to promote SAT1 pre‐mRNA splicing, thereby increasing proliferative and metastatic potential [270]. Hormone receptor status also shapes splicing: in ER⁺ breast cancer, the PML1 isoform cooperates with WDR5 to regulate stemness‐related genes such as YAP1, driving proliferation, invasion, and stem‐like traits [271], while splicing factors including FMRP [272] and RAVER1 [273] modulate ferroptosis and miRNA pathways.

In gynecological cancers, dysregulated splicing factors similarly govern malignant progression via subtype‐specific mechanisms. In ovarian cancer, SRSF9 induces oncogenic NUMB isoform switching through phase separation [94]. In endometrial cancer, SF3A3 promotes proliferation and survival by regulating c‐FOS splicing [141]. Overexpression of SNRPB, linked to poor prognosis, preserves POLD1 splicing fidelity to supports metastasis [274], underscoring the critical role of splicing in gynecological malignancies.

Neurological tumors, including glioblastoma, exploit splicing alterations to maintain stemness and modulate the immune microenvironments. For instance, in SHH‐medulloblastoma, EFTUD2 upregulation triggers KIF3A exon skipping, therapy potentiating GLI2 activity and downstream signaling [147]. OTX2 sustains a protumor stem cell splicing program (e.g., PPHLN1 splicing) and drives group 3 medulloblastoma progression [275]. In glioblastoma, the macrophage‐specific MS4A7‐s isoform drives M2 polarization via PI3K/AKT signaling [276]. Stress‐induced tDDX3X‐C facilitates tumor adaptation through loss‐of‐function PRDM2 splicing [215], while PTBP1 rewires metabolism and signaling via CERS5 and MPZL1 isoform switching [258]. In meningioma, SRSF1‐driven splicing predict recurrence and offers targets for ASO [277]. Conversely, PTBP2 inhibits IRF9 splicing in neuroblastoma to amplify type I interferon signaling, recruiting immune effectors and improving prognosis [278]. Therapeutic targeting of splicing machinery—for example, the SF3B1–PHF5A complex—shows promise in selective killing of neural crest tumor cells [279]. In glioblastoma, promoter hypomethylation upregulates the lncRNA HSD52, which stabilizes the NONO/SFPQ complex and alters splicing programs to confer chemoresistance [280].

AS dysregulation also influences progression and therapy resistance in other solid tumors. In papillary thyroid cancer, QKI downregulation leads to an oncogenic long isoform of E‐Syt2 (E‐Syt2L), promoting growth and metastasis [281]. In anaplastic thyroid cancer, NSUN2 regulates SRSF6 splicing to induce multidrug resistance [282]. In nasopharyngeal carcinoma, EBNA1 interacts with SRSF1 via phase separation to alter SRRM1 splicing and promote tumorigenesis [122]. In Ewing sarcoma, DHX9 recruits U2 snRNP/SF3B1 to regulate CTTN splicing, affecting cell migration and invasion [283].

#### 4.2.2 Hematologic Malignancies

Recurrent mutations in splicing factors drive oncogenesis in hematologic cancers. In AML, SRSF2 (e.g., P95H) and SF3B1 (e.g., K700E) mutations alter RNA binding or branchpoint recognition, leading to oncogenic splicing (e.g., BRCA1 exon skipping) and genomic instability [256, 257]. Mutations in S100A9 and cohesin are associated with specific AS patterns in AML [284]. In multiple myeloma, aberrant NHEJ, structural variations, and increased AS frequency form a high‐risk axis; aberrant splicing of FCRL5 and CRBN contributes to drug resistance and poor prognosis [285, 286]. A large‐scale transcriptomic analysis of 3760 hematologic malignancy samples revealed an average of two aberrantly spliced genes per sample and identified a truncated LRP1B transcript specifically expressed in hairy cell leukemia variant, suggesting its biomarker potential [287]. A heterozygous CEBPA mutation disrupting the bZIP domain—in a background of RUNX1 and SRSF2 mutations—drives MDS progression [288]. These findings highlight the prevalence of splicing defects in blood cancers and their diagnostic and therapeutic relevance.

#### 4.2.3 Therapy Resistance

Therapy resistance mediated by AS is a major clinical challenge across cancer types. Chemoresistance arises via altered drug metabolism, DNA repair, and apoptosis pathways. In platinum resistance: SF3A2‐induced MKRN1‐T1 inhibits apoptosis by degrading FADD [172]; SNRPA modulates ERCC1 splicing to enhance DNA repair [289]; PTBP1 activates JNK signaling via the circATIC/miR‐1247‐5p/RCC2 axis [290]; and SRSF9‐mediated SLC37A4 exon skipping yields a truncated protein [56]. In anthracycline resistance, the NCOR2 splice variant (BQ323636.1) activates NRF2 to reduce ROS levels [291], while CCND1b accelerates the cell cycle to mitigate drug toxicity [269]. SF3A3 rewires c‐Fos splicing to engage antiapoptotic pathways [141], whereas RBM25 dysfunction reduces proapoptotic Bcl‐xS and promotes multidrug resistance [292]. Impaired macroH2A1.1 splicing heightens sensitivity to TOP1 inhibitors [132]. LINC01852 diminishes chemoresistance by blocking SRSF5‐mediated PKM splicing and metabolic reprogramming [293]. In lung cancer, AKT‐mediated phosphorylation of HNRNP L inhibits its binding to ESS elements, promoting expression of antiapoptotic caspase‐9b isoform and conferring chemoresistance [51]. Cyperotundone enhances chemosensitivity in breast cancer via SRSF1 [47].

Targeted therapy resistance often results from isoforms lacking drug‐binding domains or activating alternative pathways. In ERBB2‐targeted therapy: HER2 Δ16 evades trastuzumab binding and constitutively activates signaling [294]; the ERBB2 i14e isoform causes trastuzumab resistance in gallbladder cancer, which is reversible by ASOs [57]; an ERBB2 deletion (c.644‐66_‐2del) leads to loss of the pertuzumab‐binding domain in metastatic CRC [295]. In EGFR inhibition, RON splice variants (Δ155, Δ160, Δ165) drive progression and cetuximab resistance in CRC; the inhibitor WM‐S1‐030 shows efficacy [296]. In B‐ALL, NT5C2 splicing variants confer gain‐of‐function mutations leading to 6‐mercaptopurine resistance but sensitivity to mizoribine [178]. In anaplastic thyroid cancer, NSUN2 regulates SRSF6 splicing to enhance UAP1 and ABC transporter function, inducing multidrug resistance reversible by NSUN2 targeting [282].

Immunotherapy resistance involves altered immune checkpoint expression, immune cell function, and antigen presentation. In the PD‐1/PD‐L1 axis: the soluble PD‐L1 Δ3 isoform acts as a decoy receptor [297], and the PD‐1^28 isoform in T cells exerts immunosuppression [235]. GSDMB splice variants modulate pyroptosis and antitumor immunity [237]; PTBP3‐generated IL‐18 Δ inhibits PD‐1 degradation in CD8⁺ T cells, promoting immune escape [238]. Aberrant splicing can also generate immunogenic neoantigens activating CD8⁺ T cells [298], and hMENA isoforms influence immune contexture and ICB response via tertiary lymphoid structure formation [299]. In B‐cell malignancies, a shift from CD20‐V3 to V1 splicing reduces protein expression and confers resistance to mosunetuzumab [236]. AS of HLA genes is a common immune evasion mechanism in lung adenocarcinoma and squamous cell carcinoma [300, 301]. HNRNP L‐mediated EIF4G1 splicing counteracts immune checkpoint blockade resistance in castration‐resistant prostate cancer [302].

In radiotherapy resistance, PTBP1 promotes switching from DNMT3B‐S to DNMT3B‐L, suppressing DUSP2 and enhancing radioresistance in prostate cancer [303]. TXNL4B, interacting with PRP3, regulates FANCI splicing to produce radioresistant FANCI‐12/13 isoforms; PRP3 inhibition sensitizes lung cancer cells to radiation [244]. In endocrine resistance, in ER⁺ breast cancer, PML1 isoform expression confers resistance to fulvestrant [271].

In summary, aberrant AS plays a central role in cancer development, progression, and therapy resistance. It contributes extensively to tumor‐specific signaling reprogramming and modulates response to diverse treatments, highlighting its potential as both a mechanistic hub and a therapeutic target.

### 4.3 Neurological Disorders

The brain has the most intricate AS landscape in the human body, and neurodevelopment and functional maintenance depend on its exact regulation. Numerous neurological conditions are closely linked to dysregulation of splicing [10].

#### 4.3.1 Neurodegenerative Diseases

Widespread dysregulation of AS represents a common early pathological feature across multiple neurodegenerative conditions. In spinocerebellar ataxias [304] and Huntington's disease [305], aberrant splicing of genes involved in ion channel and synaptic function occurs early and correlates with disease progression. AD is characterized by a genome‐wide decline in splicing fidelity, including mis‐splicing of PSEN2 [12, 13]. In the hippocampus, AS of Apoer2 generates an intracellular fragment that influences energy metabolism and signaling; its dysregulation is associated with neurodegeneration in AD [306]. In ALS, pronounced splicing alterations are observed across several brain regions, most notably the cerebellum, suggesting pathology beyond the motor system [14]. Genetic evidence further indicates that intronic variants contribute to ALS pathogenesis by driving erroneous splicing of genes such as PCP4 [307]. Similarly, an aberrantly spliced isoform of SFPQ (altSFPQ) exhibits reduced phase separation capacity and mislocalization in ALS [308]. In multiple sclerosis, dysfunctional hnRNP A1 leads to broad splicing defects in genes critical for neuronal function, thereby promoting neurodegeneration [309].

#### 4.3.2 Neurodevelopmental Disorders

Genetic defects in core spliceosomal components constitute an important class of pathogenic mutations underlying NDDs. Noncanonical splicing variants are significantly enriched in NDD patients and contribute to disease by mis‐splicing genes involved in glutamatergic synapses and other key neural processes [310]. Erroneous microexon splicing leads to abnormal aggregation of CPEB4, disrupting translational control in autism spectrum disorder (ASD) [311]. Loss PTEN lead to broad splicing dysregulation of multiple ASD risk genes [312]. Targeting splicing regulation offers novel therapeutic avenues; for example, modulating PTBP2 corrects the mis‐splicing of SYNGAP1 and restores its protein expression [182]. Additionally, ATRX regulates neuronal differentiation through phase separation, and its disruption impairs neurodevelopment [112]. Differential phase behavior among SNCA (α‐synuclein) splice isoforms may also contribute to the pathologies associated with Parkinson's disease [313].

Heterozygous mutations in the spliceosomal gene SNW1 disrupt interactions with core spliceosome proteins, resulting in widespread splicing defects, impaired neural stem cell proliferation, and increased apoptosis [198]. De novo variants in the splicing regulators U2AF2 and PRPF19 similarly cause splicing dysregulation and reduced neurogenesis, operating within regulatory networks involving RBFOX1 to influence brain development [314]. Distinct mis‐splicing patterns are produced by dominant mutations in major snRNA genes, such as RNU4‐2, RNU5B‐1, and RNU5A‐1, and their severity is correlated with clinical presentation [315]. Protein stability and splicing function are compromised by biallelic variants in RBM42, resulting in multisystem disorders with severe neurological abnormalities [316]. Together, these findings emphasize that functional integrity of the core spliceosome is crucial to healthy neurodevelopment.

#### 4.3.3 Psychiatric Disorders

As a vital link between genetic susceptibility and environmental risk, AS is important in psychiatric disorders. Significant splicing reprogramming is induced by psychological stress in the mouse brain. Differentially spliced genes are enriched in neural pathways, and their human homologs are linked to a number of psychiatric disorders, indicating that splicing changes may mediate environmental contributions to disease risk [201]. The functional significance of splicing in psychiatric pathogenesis is confirmed by genetic studies that show certain splicing events in genes like ELOVL7 are associated with alcohol use disorder and correlated with structural changes in the brain [317]. Pleiotropic genes, such as the splicing regulator RBM6, are also identified by large‐scale genetic analyses as key participants in metabolic and psychiatric comorbidities, indicating a common mechanistic basis through splicing regulation [318].

### 4.4 Cardiovascular Diseases

Precise control of AS is essential for maintaining cardiac structure and function, and its disruption is increasingly recognized as a major contributor to diverse cardiovascular diseases [203, 204, 205]. In heart failure and cardiomyopathy, RBPMS cooperates with RBM20 to regulate splicing of sarcomeric genes such as TTN, safeguarding contractile integrity [208], while DDX5 maintains calcium homeostasis through CAMK2D splicing [204], and loss of QKI results in profound sarcomere disarray and heart failure [205]. Long noncoding transcripts, including TTN‐AS1‐276, further modulate sarcomere properties by influencing TTN isoform expression [319]. Splicing dysregulation also drives fibrotic remodeling, exemplified by RBMS1‐dependent alterations in LMO7 splicing that activate the TGF‐β1 pathway [320]. In congenital heart disease, endothelial PTBP1 deficiency induces left ventricular noncompaction through aberrant splicing of ARRB1 [203]. Drug‐induced cardiotoxicity can similarly arise from splicing defects, as illustrated by sorafenib‐mediated RBM20 inhibition and mis‐splicing of mitochondrial regulators such as SLC25A3 [225]. In coronary artery disease, SRSF1 enhances NF‐κB activation via ENPP3 splicing, amplifying myocardial inflammation [50]. Arrhythmias frequently result from altered splicing of ion channel genes, including SCN5A and CACNA1C, and high‐throughput platforms such as ParSE‐seq reveal that many pathogenic variants exert their effects by disrupting splicing [321, 322]. Together, these findings underscore AS as a key mechanistic axis in cardiovascular pathology and highlight splicing regulators and networks as promising therapeutic targets.

### 4.5 Autoimmune and Inflammatory Diseases

AS plays a central role in autoimmune and inflammatory diseases by regulating immune cell activation, cytokine signaling, and antigen presentation, often in a cell‐type and population‐specific manner.

In systemic lupus erythematosus, fluctuations in disease activity coincide with widespread transcriptional and splicing reprogramming across pathways such as TLR, BTK, CTLA‐4, and NLRP3 [323, 324], accompanied by interferon signatures that stratify clinical subtypes [325], distinct epigenetic and transcriptomic states in monocyte subsets [326], and enhanced survival programs in antibody‐secreting cells that sustain autoantibody production [327]. Additional regulatory layers include exosomal miR‐122‐5p, which promotes M1 macrophage polarization through the FOXO3–NF‐κB axis and exacerbates lupus nephritis [328], and skewed X‐chromosome inactivation linked to interferon signaling, offering new avenues for disease assessment [329].

In rheumatoid arthritis, loss of RBM25 drives ACLY exon skipping, boosting glycolysis and acetyl‐CoA production to fuel macrophage‐mediated inflammation [330]. while clinical pain severity correlates with splicing variation in HLA‐DRB1 and TNF [331]. Splicing also contributes to inflammatory pathology in other tissues: a soluble IL1RN isoform acts as an inflammatory checkpoint in KRAS‐mutant cholangiocarcinoma and predicts improved anti‐PD‐1 responsiveness [233], whereas in Graves’ disease, a population‐specific sQTL alters TCHP splicing and illustrates ancestry‐linked variation in splicing‐mediated susceptibility [240]. Together, these findings highlight AS as a key regulator of autoimmune pathogenesis and a potential source of therapeutic leverage.

### 4.6 Metabolic Diseases

AS is a central regulator of metabolic homeostasis, shaping the balance of isoforms that govern metabolic enzymes, hormone receptors, and signaling pathways. Its dysregulation is increasingly recognized as a driver of obesity, type 2 diabetes, and nonalcoholic fatty liver disease [332].

In diabetes, defective splicing perturbs insulin secretion, β‐cell function, immune tolerance, and end‐organ integrity. RBFOX2 controls splicing of insulin granule‐related genes [333], SOX9 loss causes widespread mis‐splicing in β‐cells [170], and HuR enhances insulin sensitivity by modulating insulin receptor isoforms [334]. Additional regulators, including RBM25 and WFS1, modulate apoptotic pathways through BCL2L1 [292] and WFS1 splicing [175]. In autoimmunity, insulin splice variants in δ‐cells can activate cytotoxic T cells [335], and interferon‐α promotes alternative epitope presentation in inflammatory settings [336]. Diabetic complications further reflect splicing defects, with RBFOX2‐mediated CACNA1C mis‐splicing contributing to cardiomyocyte calcium overload [337] and the lncRNA evf‐2 promoting podocyte injury through altered cell‐cycle gene splicing [338].

Hepatic metabolic disease similarly involves splicing‐driven pathway rewiring: minor intron retention in INSIG1/2 activates SREBP1c and lipogenesis [339]. SRPK2‐dependent inhibition of SRSF10 disrupts LPIN1 splicing to drive steatosis [216], and CUG repeat expansions in myotonic dystrophy lead to ACACA mis‐splicing and lipid accumulation [340].

In obesity, SRSF1 maintains mitochondrial function and thermogenesis through NDUFS3 splicing [48], whereas SRSF3 deficiency downregulates CRIg, promoting inflammation and insulin resistance [341]. An intracellular isoform of uromodulin further supports metabolic resilience by increasing NAD⁺ and ATP levels in renal tubular cells [143]. Together, these findings underscore the pervasive impact of splicing regulation across metabolic tissues and its potential as a therapeutic entry point for metabolic disease.

### 4.7 Respiratory Diseases

AS is increasingly implicated in the pathogenesis of respiratory diseases, including pulmonary hypertension, fibrosis, and chronic obstructive pulmonary disease (COPD). In pulmonary hypertension, the super‐enhancer‐driven lncRNA HCG20 binds and stabilizes U2AF2, promoting aberrant splicing of EIF2AK4 and contributing to endothelial dysfunction [140]. Pulmonary fibrosis is similarly shaped by splicing‐mediated metabolic shifts, exemplified by SRSF7‐dependent PKM isoform changes that drive fibroblast activation [209]. Genetic susceptibility to COPD can also arise from splicing alterations, as illustrated by the rs34712979‐A variant, which introduces a cryptic acceptor site in NPNT and disrupts protein architecture [342]. Mendelian randomization analyses further suggest shared genetic mechanisms between splicing events in ATP11A and DPP9 and the severity of COVID‐19 and idiopathic pulmonary fibrosis, while MUC1 splicing variants have been linked to COVID‐19 susceptibility [211]. Together, these findings underscore the central role of RNA splicing and RBPs in respiratory disease pathogenesis and highlight their potential as therapeutic targets [5].

### 4.8 Other Diseases

Splicing dysregulation contributes to a wide array of human disorders by perturbing key genetic and molecular pathways. Large‐scale studies reveal that noncoding variants can act as sQTLs, modulating disease risk through altered splicing of critical genes [343]. In reproductive disorders, the hnRNPC–HuR complex maintains correct splicing of meiotic genes such as SYCP1 and BRCA1; and its disruption leads to meiotic arrest and infertility [52]. Systemic sQTL analyses further link splicing defects to hypertension (WARS1), dermatitis (IL7R), and COVID‐19 susceptibility (IFNAR2), highlighting novel therapeutic targets [343]. Developmental abnormalities also arise from splicing misregulation: aberrant methylation of MSX1 by PRMT1 disrupts phase separation, forming pathological gels that impair palatal development in cleft palate [344]. In muscular disorders, ASOs inducing exon skipping in Duchenne muscular dystrophy create PTCs, triggering transcriptional adaptation and compensatory utrophin upregulation [15], while hnRNPs contribute to limb‐girdle muscular dystrophy, myotonic dystrophy type 1 (DM1), oculopharyngeal muscular dystrophy, sporadic inclusion body myositis, multisystem proteinopathy, and spinal muscular atrophy (SMA) [345]. Mutations in GPATCH11 cause loss of its G‐patch domain and mislocalization, leading to retinal dystrophy [346], and in myotonic dystrophy, sequestration of MBNL proteins in nuclear aggregates disrupts splicing, miRNA processing, and skeletal muscle function [150]. Collectively, these examples illustrate the pervasive role of splicing dysregulation across diverse human diseases, emphasizing its importance as a central pathogenic mechanism.

### 4.9 Concluding Perspectives

Dysregulated AS is a central mechanism in human disease, contributing to pathogenesis through functional isoform imbalances across cancer, neurological, cardiovascular, autoimmune, metabolic, and respiratory disorders. These insights not only establish splicing as a critical molecular node in disease but also highlight its therapeutic potential. Strategies targeting splicing, such as modulating splicing factor activity, exon‐specific interventions, small molecule inhibitors, and antisense technologies, offer promising avenues for treating a wide range of refractory conditions.

## 5 Therapeutic Targeting of AS

As explained in Section 4, the widespread dysregulation of AS in human diseases has spurred the creation of therapeutic approaches meant to correct pathogenic splicing events. To restore physiological isoform balance, these interventions operate at multiple levels, either directly reprogramming pre‐mRNA splicing, targeting the core spliceosome, or modifying particular regulatory factors [9, 347].

The therapeutic landscape for splicing defects is examined in this section with a focus on four main modalities: CRISPR/dCas13 editing, ASOs, SMSMs, and neoantigen‐based immunotherapies. Referencing approved therapies like nusinersen and eteplirsen as well as an overview of current trials, we assess the clinical trajectory of these strategies. Lastly, we address critical barriers, including resistance, delivery, and specificity, and we suggest future paths for the field.

### 5.1 Small Molecule Splicing Modulators

With the benefit of oral bioavailability and systemic distribution, SMSMs represent a promising class of pharmacologic agents that target splicing machinery components.

#### 5.1.1 Targeting Core Spliceosomal Components

The spliceosome assembly node SF3B represents a prime therapeutic target. SF3B1 inhibitors, such as E7107, have strong antitumor effects by suppressing oncogenic isoforms like RON Δ160 and concurrently promoting proapoptotic exon inclusion like MCL1 to MCL‐1S [174]. Distinct from SF3B targeting, the oral CDC‐like kinase (CLK) inhibitor BH‐30236 is now advancing through Phase I/Ib trials for refractory AML (NCT06501196), representing a parallel strategy to modulate splicing via kinase inhibition. Synergistic approaches are essential in solid tumors, where somatic spliceosomal mutations are less common. For instance, the HDAC2 inhibitor romidepsin effectively resensitizes hepatocellular carcinoma cells to PARP inhibitors by correcting pathological BRCA1 splicing through SmD2 hyperacetylation [348]. Radiation sensitivity can be successfully restored by blocking the TXNL4B–PRP3 complex, which mediates aberrant FANCI splicing that confers radioresistance in lung cance [244].

#### 5.1.2 Targeting Splicing Regulatory Kinases and Phase Separation

Kinases that phosphorylate SR proteins and other splicing factors are appealing druggable nodes outside of the core spliceosome. SRPK2 activation promotes pathogenic LPIN1 splicing, to the lipogenic lipin‐1β isoform via SRSF10, in alcohol‐associated liver disease; genetic or pharmacological inhibition of SRPK2 reduces hepatic steatosis [216]. High‐affinity CLK4 inhibitors reprogram oncogenic splicing networks in pancreatic ductal adenocarcinoma, by modifying SRSF4/SRSF6, causing cell cycle arrest and apoptosis [349]. This strategy is beginning to receive clinical validation, as evidenced by the PRMT5 inhibitor GSK3326595 [350]. This medication causes total cell cycle arrest in patients with early‐stage breast cancer by changing MDM4 splicing (NCT04676516).

Although it remains challenging, disrupting pathological condensates offers a novel therapeutic approach given the prominent role of aberrant LLPS in driving diseases (Sections 4.1, 4.2, 4.3, and 4.8). In oral squamous cell carcinoma, the splicing factor SRSF9 undergoes aberrant LLPS (as detailed in Section 2.4), which drives pathogenic exon skipping in the SLC37A4 transcript. This event produces a truncated protein isoform that promotes tumor growth and progression [56]. Disrupting the phase separation capability of such factors could potentially reverse aberrant splicing. Despite substantial basic research underscoring the pivotal role and therapeutic potential of phase separation in diseases, particularly tumors and neurodegenerative disorders, few successful clinical translations have emerged to date.

### 5.2 Antisense Oligonucleotides

ASOs are short, synthetic nucleic acids that hybridize to pre‐mRNA via Watson–Crick base pairing, sterically blocking splicing factors binding and influencing splice site selection. They are perfect for targeting individual pathogenic splicing events due to their high specificity and programmability.

#### 5.2.1 Correction of Pathogenic Splicing in Genetic Diseases and Cancer

The most well‐known application of ASO therapy is in the treatment of genetic neuromuscular disorders. Eteplirsen, approved by the United States Food and Drug Administration (US FDA) in 2016, induces skipping of exon 51 in the DMD gene to restore the reading frame and produce a functional, albeit truncated, dystrophin protein, attenuating disease progression in DMD [351, 352]. Similarly, nusinersen (Spinraza), approved in 2016, encourages the SMN2 transcript to include exon 7, significantly raising the levels of functional SMN protein and enhancing motor function and survival in cases of SMA [353, 354]. With drugs like DYNE‐101 for DM1 (NCT05481879) and ONB‐CFTR for cystic fibrosis (NCT05100823) currently progressing in clinical trials, the ASO pipeline is expanding beyond proven successes.

ASOs can be used in oncology to target oncogenic splicing events or factors. Silencing of the splicing factor SNRPB with ASOs in endometrial cancer induces aberrant retention of an intron in the *POLD1* transcript, resulting in a PTC and degradation of the message, thereby inhibiting tumor proliferation [274]. Another strategy involves forcing the inclusion of a poison exon within the transcript of an essential splicing factor itself. For example, ASOs that promote the inclusion of a toxic exon in *TRA2β* trigger its degradation, exerting antitumor effects [355]. Additionally, ASOs can also be designed to counteract specific splicing variants that drive therapy resistance. A shortened protein produced by PTBP3‐triggered exon skipping in COX11 allows cancer cells to evade cuprotosis in gastric cancer. When combined with copper ionophores, ASOs that target this event efficiently cause cell death [146]. Furthermore, in non‐small cell lung cancer, customized ASOs can specifically suppress oncogenic splice variants of *EGFR*, such as L858R/T790M, presenting a strategy to overcome tyrosine kinase inhibitor resistance [356].

#### 5.2.2 Generating Neoantigens and Modulating the Immune Microenvironment

Splicing interventions offer innovative approaches to enhance immune surveillance. Tumor‐specific neoantigens can be induced by ASOs; for instance, an ASO‐mediated shift in CD20 splicing generates a Δ‐CD20 isoform that triggers cytotoxic T‐cell responses, possibly overcoming Rituximab resistance in B‐cell lymphoma (NCT02844491) [236]. An immunosuppressive microenvironment can also be reversed by targeting noncoding RNAs that regulate splicing. An ASO targets the circular RNA *cALG8* in pancreatic cancer, interferes with its ability to facilitate SRSF7 phosphorylation and the translation of an oncogenic *ATM* splice variant, making the tumors more sensitizing to chemotherapy and anti‐PD‐1 immunotherapy [357]. Similarly, inhibition of the splicing factor *SNRNP200* in TNBC activates CD8⁺ T‐cell infiltration and reverses its glycolytic splicing program, synergizing with PD‐1 checkpoint blockade [358].

### 5.3 Gene Editing and CRISPR‐Based Technologies

CRISPR systems offer the potential for permanent correction of disease‐causing splicing defects by directly editing the genome or by transiently reprogramming RNA splicing.

#### 5.3.1 dCas13‐Mediated Precise Splicing Reprogramming

CRISPR systems that target catalytically dead RNA, such as dCas13/dCasRx, allow for precise control of splicing results without modifying the DNA sequence. The dCas13/gRNA complex acts primarily through steric hindrance, binding to SREs on the pre‐mRNA to block the recruitment of splicing factors and alter splice site choice [359]. For instance, the SMN2 gene's splicing pattern has been successfully switched using dCasRx, providing a programmable platform for basic research and therapeutic exploration [164]. Effector domains can be fused with dCas13 to increase flexibility and efficiency. A fusion protein of dCasRx and the splicing activator RBM25 can robustly activate approximately 90% of endogenous alternative exons and allows for the simultaneous regulation of multiple exons via a gRNA array, enabling the functional dissection of complex splicing networks [360]. The identification of functional SREs across the entire genome is rendered by high‐throughput screening platforms like SpliceRUSH, which combine dCas13 with gRNA libraries. This approach can directly inform the optimal design of therapeutic ASOs, uncover novel regulatory elements [359].

#### 5.3.2 Therapeutic Potential in Various Diseases

CRISPR/Cas9‐mediated genome editing holds promise for correcting splicing mutations at their source. Repairing a pathogenic splice‐site mutation in the *WFS1* gene prevents β‐cell apoptosis and restores the normal transcriptome in wolfram syndrome [175]. By correcting disease‐causing mutations in *RBM20* that disrupt its nuclear localization and splicing regulatory function, CRISPR strategies in DCM aim to restoring splicing of titin (*TTN*) and other sarcomeric genes [361, 362]. CRISPR screening can identify novel synthetic lethal interactions. *SNRPA* knockout enhancing cisplatin sensitivity in lung adenocarcinoma by reversing its mediated aberrant splicing of *ERCC1* exon 8 [289]. Targeting regulatory noncoding RNAs can mitigate pathological splicing; in pulmonary hypertension, intervention against the super‐enhancer‐driven lncRNA *HCG20* corrects its pathogenic splicing of *EIF2AK2 by* stabilization U2AF2 [140].

### 5.4 Immunotherapy and Splicing Regulation

The interplay between AS and antitumor immunity offers fertile ground for therapeutic innovation, primarily through two mechanisms: generating neoantigens and directly modulating the immune microenvironment.

Neoantigens that can be exploited for immunotherapy are abundant in tumor‐specific splicing events. Widespread neojunctions, or unique splice junctions do not present in normal tissues, are frequently seen in cancers and can result in immunogenic peptides capable of activating cytotoxic T cells [298]. Strategies using small molecules, ASOs, or CRISPR to induce or enhance the expression of these neoantigens are under active investigation to boost immune recognition of tumors [347].

Splicing factors themselves play a direct role in shaping the tumor immune landscape. For instance, the loss of GPATCH3 reduces DHX15's ATPase activity, which causes immune‐related genes like CD44 and CXCR3 to splice abnormally. This results in a 67% reduction in CD8⁺ T cell infiltration into tumors and confers resistance to anti‐PD‐1 therapy [363]. Conversely, PRMT5 inhibitors improve the effectiveness of immune checkpoint blockade by changing splicing, increasing MHC‐II expression, and encouraging T‐cell infiltration [349].

Splicing immune cell receptors and ligands is a crucial immune evasion mechanism. Tumors can promote the expression of immunosuppressive splice variants of immune checkpoints. For instance, a soluble PD‐L1 Δ3 isoform functions as a decoy receptor [297], and a specific PD‐1 splice variant (PD‐1^28) in T cells exerts potent immunosuppressive effects [235]. A exon in IL‐18 pre‐mRNA is skipping by PTBP3‐mediated in gallbladder cancer, resulting in a truncated ΔIL‐18 protein that stabilizes PD‐1 on T‐cells, impeding their function and encouraging immune escape [238]. Moreover, the sensitivity of tumor cells to granzyme A‐mediated pyroptosis is determined by AS of *GSDMB*. Tumors often express inactive GSDMB isoforms (1/2/5) to evade this lytic cell death, while ASOs that promote the expression of functional isoforms (3/4) can resensitize them to T‐cell killing [237].

### 5.5 Clinical Translation and Approved Therapies

The US FDA has granted landmark approvals to splicing‐targeted therapies which proved their potential for medical use. Nusinersen (Spinraza) was approved in 2016 as an ASO which enhances SMN2 exon 7 inclusion to produce functional SMN protein. The new treatment method produces exceptional results because it improves motor abilities and extends survival time for SMA patients [353, 354]. Similarly, eteplirsen (2016) induces *DMD* exon 51 skipping to regenerate functional dystrophin and slow disease progression in Duchenne muscular dystrophy [351, 352]. Risdiplam (Evrysdi; 2020), an orally bioavailable small molecule splicing modifier, provided a systemic alternative for SMA, yielding survival benefits superior to historical controls [364, 365].

These clinical milestones have catalyzed a diverse pipeline of splicing‐targeted therapies across oncology and neurology (Table 3).

**TABLE 3:** Clinical trials targeting aberrant alternative splicing.

Disease area	NCT number	Phase	Target/mechanism /therapeutic agent	Status	Enrollment	Splicing target
AML, HR‐MDS	NCT06501196	1/1b	CLK inhibitor modulating RNA splicing; BH‐30236 (CLKi)	Recruiting	74	SRSFs
DM1	NCT05481879	1/2	DMPK CUG repeats splicing; DYNE‐101 (Dyne Tf)	Recruiting	116	DMPK
Breast cancer	NCT04676516	2	PRMT5i, MDM4 splicing; GSK3326595 (PRMT5i)	Completed	40	MDM4
Solid tumors and non‐Hodgkin's lymphoma	NCT02783300	1b	PRMT5i, MDM4 splicing; GSK3326595 (PRMT5i)	Completed	297	MDM4
Solid tumors	NCT03355066	1	Splicing modulator; SM08502 (CLK/DYRKi)	Terminated^a^	82	Global splicing
B‐ALL/B‐LBL	NCT02981628	2	CD22 splicing and resistance; InO (ADC)	Recruiting	80	CD22
Huntington's disease	NCT05111249	2	Mutant huntingtin protein; Branaplam	Terminated^a^	26	HTT, SMN2
B‐cell lymphoma	NCT02844491	NA	Δ‐CD20 expression; rituximab (anti‐CD20)	Terminated	28	MS4A1 (CD20)

Data for this table were obtained from ClinicalTrials.gov (https://clinicaltrials.gov/).

*Abbreviations*: ADC: antibody–drug conjugate; AML: acute myeloid leukemia; B‐LBL: B‐cell lymphoblastic lymphoma; CLL/ALL: chronic lymphocytic leukemia/acute lymphoblastic leukemia (includes B‐ALL, T‐ALL); DM1: myotonic dystrophy type 1; mAb: monoclonal antibody; NA: not applicable (phase); Tf: targeted therapeutics payload delivery platform; Δ‐CD20: exon‐skipped CD20 isoform.

^a^Trial terminated potentially due to toxicity or lack of efficacy.

### 5.6 Challenges and Future Perspectives

The clinical translation of splicing‐targeted therapies, although increasingly promising, faces several formidable yet tractable challenges, and lessons from pioneering agents such as nusinersen provide a valuable framework for future progress. A central obstacle is achieving the specificity required to avoid off‐target toxicity arising from disruption of physiological spliceosome function. First‐generation SMSMs that act on core factors such as SF3B1 frequently exhibit narrow therapeutic windows due to global splicing inhibition, whereas nusinersen's exon‐specific approach illustrates how targeting a single cis‐element can deliver efficacy with an acceptable safety profile. Future SMSMs will likely need context‐dependent designs, including exploitation of synthetic‐lethal vulnerabilities in spliceosome‐mutant cancers or the development of bifunctional molecules that recruit defined splicing factors to individual transcripts.

Efficient delivery of nucleic acid‐based therapeutics beyond the central nervous system and liver remains another major barrier. Although intrathecal dosing enabled nusinersen's clinical success, systemic diseases and solid tumors will require more sophisticated delivery platforms. Next‐generation lipid nanoparticles, GalNAc conjugates for hepatocyte‐targeted delivery, engineered AAV capsids with expanded tissue tropism  and emerging exosome‐based systems offer promising routes to enhance targeting and endosomal escape.

Resistance mechanisms further complicate long‐term efficacy. Tumors can upregulate AS factors or activate bypass pathways, and even established therapies may encounter diminishing benefit due to neuronal loss or reduced intracellular uptake. Rational combination strategies provide one path forward, pairing splicing modulators with immunotherapies to boost neoantigen presentation, with epigenetic regulators to reshape the splicing landscape or with dual‐node inhibitors to block compensatory signaling.

Therapeutic success will also depend on robust biomarkers that capture patient‐specific splicing states. RNA‐seq‐derived splicing signatures, genomic profiling of splicing‐factor mutations and liquid‐biopsy detection of circulating tumor RNA offer promising tools for patient stratification and dynamic monitoring.

Looking ahead, interdisciplinary innovations will shape the next decade of therapeutic development. Integration of artificial intelligence with multiomics datasets will improve predictive modelling of splicing dysregulation and guide rational design. New therapeutic modalities—including PROTACs for targeted degradation of oncogenic splicing factors, small molecules that disrupt pathological condensates and in vivo base‐editing technologies—promise increasingly precise and durable interventions. High‐fidelity preclinical models, such as patient‐derived organoids and xenografts that preserve splicing heterogeneity, will be essential for translational validation.

Collectively, these advances mark a shift from broad spliceosome inhibition toward highly precise, RNA‐centric therapies. With continued mechanistic insight, clinical experience and technological innovation, splicing modulation is poised to become a foundational element of precision medicine across diverse refractory diseases.

## 6 Conclusion and Future Perspectives

AS stands as one of the most sophisticated and dynamic processes of gene regulation in eukaryotes, profoundly expanding the functional diversity of the proteome from a limited set of genes. The multilayered regulatory landscape of AS has been outlined in this review, ranging from the intricate assembly of the spliceosome and sequence‐specific recognition by cis‐elements and trans‐factors, to the higher order regulation mediated by RNA structures, phase separation, epigenetics, and environmental cues. We have further explored its pivotal roles in governing basic biological processes, such as cell differentiation, organ development, metabolic adaptation, and immune responses. We have also highlighted how its dysregulation serves as a common pathogenic driver in a variety of diseases including cancer, neurological disorders, and cardiovascular conditions.

The therapeutic targeting of AS has rapidly progressed from a theoretical concept to clinical practice. Strategies including SMSMs, ASOs, CRISPR/dCas‐based RNA editing, and immunotherapy are showing encouraging promise in reestablishing cellular homeostasis and repairing pathological splicing events. The successful clinical translation of splicing modulation is demonstrated by approved therapies such as nusinersen and eteplirsen, which provide hope for genetic disorders that were previously thought to be incurable.

Despite this, substantial challenges remain. Improving the specificity of therapeutic interventions is paramount to minimize off‐target effects on normal splicing. Enhancing delivery efficiency, particularly to the brain and solid tumors, requires innovations in nanocarriers, viral vectors, and tissue‐specific targeting technologies. Furthermore, the development of rational combination therapies will be required to overcome resistance mechanisms like compensatory splicing factor expression or alternative pathway activation.

Future progress will be fueled by interdisciplinary convergence. The design of isoform‐specific therapeuticsi and the predictive modeling of splicing networks will be made possible by advances in artificial intelligence and multiomics integration. Single‐cell and spatial technologies will uncover cell‐type‐specific splicing programs, improving biomarker discovery and patient stratification. Novel modalities, including PROTACs degrading splice factors, small molecules modulating phase separation, and in vivo base editing, hold great promise for achieving precise and durable corrections.

Further research is also warranted to investigation to answer several basic questions: How do physicochemical principles govern biomolecular condensates formed by splicing regulators? How does splicing reprogramming in response to stress influence long‐term disease susceptibility or transgenerational inheritance? What are the complete effects of neoantigens derived from splicing on antitumor immunity?

In summary, research on AS is transitioning from mechanistic discovery to clinical innovation. Advanced technologies from functional genomics, nucleic acid therapeutics, and artificial intelligence are paving the way toward splicing‐based diagnostics and personalized RNA medicines. Over the next decade, we anticipate that targeting the splicing landscape will yield transformative therapies for some of the most challenging human diseases, ultimately fulfilling the promise of precision RNA medicine.

## Author Contributions

Zhi‐Min Zhu was responsible for the conceptualization and Writign — original draft. Xiao‐Mei Wu was responsible for Writign – reviewing and editing. Yan Hu was responsible for Writign – reviewing and editing. Xiao‐Lan Bian was responsible for conceptualization. Ya‐Qin Wang was responsible for methodology and Writign – reviewing and editing. Qiong‐Ni Zhu was responsible for the conceptualization, methodology, Writign – reviewing and editing, and funding acquisition. All authors contributed to the article and approved the submitted version.

## Funding Information

This work was supported by Key Discipline Construction Projects of Shanghai Health System (2024ZDXK0062), Clinical Pharmacy Research Fund of China International Medical Foundation‐Chinese Society of Clinical Pharmacy (Z‐2021‐46‐2101‐2023), Shanghai Sailing Program (20YF1427000), and Ruijin Youth NSCF Cultivation Fund (2025PY112).

## Conflicts of Interest

The authors declare no conflicts of interest.

## Ethics Statement

The authors have nothing to report.

## Data Availability

The authors have nothing to report.
